# The Effect of Strength Training Methods on Middle-Distance and Long-Distance Runners’ Athletic Performance: A Systematic Review with Meta-analysis

**DOI:** 10.1007/s40279-024-02018-z

**Published:** 2024-04-17

**Authors:** Cristian Llanos-Lagos, Rodrigo Ramirez-Campillo, Jason Moran, Eduardo Sáez de Villarreal

**Affiliations:** 1 Physical Performance Sports Research Center (PPSRC), Universidad Pablo de Olavide, 41704 Seville, Spain; 2 Exercise and Rehabilitation Sciences Institute, School of Physical Therapy, Faculty of Rehabilitation Sciences, Universidad Andres Bello, Santiago, 7591538 Chile; 3 School of Sport, Rehabilitation and Exercise Sciences, University of Essex, Colchester, Essex CO43SQ UK

## Abstract

**Background:**

The running performance of middle-distance and long-distance runners is determined by factors such as maximal oxygen uptake (*V*O_2_max), velocity at *V*O_2_max (v*V*O_2_max), maximum metabolic steady state (MMSS), running economy, and sprint capacity. Strength training is a proven strategy for improving running performance in endurance runners. However, the effects of different strength training methods on the determinants of running performance are unclear.

**Objective:**

The aim of this systematic review with meta-analysis was to compare the effect of different strength training methods (e.g., high load, submaximal load, plyometric, combined) on performance (i.e., time trial and time until exhaustion) and its determinants (i.e., *V*O_2_max, v*V*O_2_max, MMSS, sprint capacity) in middle-distance and long-distance runners.

**Methods:**

A systematic search was conducted across electronic databases (Web of Science, PubMed, SPORTDiscus, SCOPUS). The search included articles indexed up to November 2022, using various keywords combined with Boolean operators. The eligibility criteria were: (1) middle- and long-distance runners, without restriction on sex or training/competitive level; (2) application of a strength training method for ≥ 3 weeks, including high load training (≥ 80% of one repetition maximum), submaximal load training (40–79% of one repetition maximum), plyometric training, and combined training (i.e., two or more methods); (3) endurance running training control group under no strength training or under strength training with low loads (< 40% of one repetition maximum); (4) running performance, *V*O_2_max, v*V*O_2_max, MMSS and/or sprint capacity measured before and after a strength training intervention program; (5) randomized and non-randomized controlled studies. The certainty of evidence was assessed using the GRADE (Grading of Recommendations Assessment, Development and Evaluation) approach. A random-effects meta-analysis and moderator analysis were performed using Comprehensive meta-analysis (version 3.3.0.70).

**Results:**

The certainty of the evidence was very low to moderate. The studies included 324 moderately trained, 272 well trained, and 298 highly trained athletes. The strength training programs were between 6 and 40 weeks duration, with one to four intervention sessions per week. High load and combined training methods induced moderate (effect size =  − 0.469, *p* = 0.029) and large effect (effect size =  − 1.035, *p* = 0.036) on running performance, respectively. While plyometric training was not found to have a significant effect (effect size =  − 0.210, *p* = 0.064). None of the training methods improved *V*O_2_max, v*V*O_2_max, MMSS, or sprint capacity (all *p* > 0.072). Moderators related to subject (i.e., sex, age, body mass, height, *V*O_2_max, performance level, and strength training experience) and intervention (i.e., weeks, sessions per week and total sessions) characteristics had no effect on running performance variables or its determinants (all *p* > 0.166).

**Conclusions:**

Strength training with high loads can improve performance (i.e., time trial, time to exhaustion) in middle-distance and long-distance runners. A greater improvement may be obtained when two or more strength training methods (i.e., high load training, submaximal load training and/or plyometric training) are combined, although with trivial effects on *V*O_2_max, v*V*O_2_max, MMSS, or sprint capacity.

**Supplementary Information:**

The online version contains supplementary material available at 10.1007/s40279-024-02018-z.

## Key Points

**Table:** 

Strength training with high loads (≥ 80% of one repetition maximum) can improve time trial and time to exhaustion running performance.
The combination in a program of two or more strength training methods (i.e., high loads, submaximal loads [40–79% of one repetition maximum], and/or plyometric training) may induce greater running performance improvement compared with one method alone.
Maximal oxygen consumption, velocity at maximal oxygen consumption, maximum metabolic steady state, and sprint capacity exhibited trivial changes after strength training.
The results are based on 38 studies and 894 (651 male individuals and 243 female individuals) middle-distance and long-distance runners, aged between 17 and 40 years, with a very low to moderate certainty of evidence.

## Introduction

In middle-distance (800–3000 m) and long-distance running (5000 m to marathon) races, performance is determined by factors such as maximal oxygen uptake (*V*O_2_max), velocity at *V*O_2_max (v*V*O_2_max), maximum metabolic steady state (MMSS), running economy [1–3], and sprint capacity [4]. Indeed, *V*O_2_max has long been used as a primary measure of an individual’s cardiorespiratory fitness, and as a marker of training effect [5]. The interplay between *V*O_2_max and running economy determines v*V*O_2_max [2, 6], whereas the MMSS (e.g., second lactate threshold) establishes the limit of steady-state muscle metabolism [20]. Running economy, defined as the amount of energy required for running at submaximal speeds [7], may differentiate running performance in athletes with similar *V*O_2_max levels [8], and sprint capacity can influence races that require changes of pace [9] or a final sprint [4].

The implementation of strength training (ST) can improve the performance in middle-distance and long-distance runners [10–14]. However, previous meta-analyses have focused mainly on running economy [11–13] and time trial running performance [13], without exploring the effects of ST on other determinants of performance (i.e., *V*O_2_max, v*V*O_2_max, and sprint capacity). For example, it has been found that ST could induce a trivial effect on *V*O_2_max in endurance athletes [15]. In addition, the incorporation of diverse ST methods has demonstrated improvements in running economy among endurance runners [10–13, 16]. Moreover, ST may improve anaerobic and neuromuscular characteristics (e.g., sprint capacity) [3]. These changes may be manifested in factors influenced by these variables, such as v*V*O_2_max [2, 6].

Strength training is a versatile method of exercise that can be customized by the manipulation of factors such as intensity, volume, inter-set rest, frequency, type and sequence of exercise, and speed of movement [17]. For instance, by manipulating the load (i.e., intensity) ST may be classified as high load training (HL, i.e., ≥ 80% of 1 repetition maximum [1RM]), submaximal load training (SubL, i.e., 40–79% 1RM), or plyometric training (PL, i.e., jump-based training with light or no loads) [18, 19]. Each of these ST methods target a specific outcome such as maximal strength, strength at submaximal loads with higher speed of movements, or stretch–shortening cycle and muscle–tendon stiffness, respectively [18]. Therefore, the effect of ST on performance and its determinants may vary depending on the specific characteristics of each ST method [14, 19]. For example, ST has shown improvements in fixed blood lactate after PL [20] and blood lactate concentration at 16 km/h after a combined HL and PL intervention [21]. However, some studies have not shown any improvement in MMSS [22–24].

The concerns described above may be related to the small number of studies that have compared ST methods, with most studies simply comparing standard running training protocols to ST. A comparison of different ST methods can entail highly complex logistical planning for researchers, meaning it is not always feasible to carry out. However, a systematic review with meta-analyses may offer a viable alternative to addressing such methodological challenges by combining studies that utilise different ST methods, thus enabling their comparison. Although some systematic reviews with meta-analyses have been published involving runners [10–14], a more comprehensive understanding of the effects of ST methods on endurance running performance (i.e., time trial and time to exhaustion) and its other determinants (e.g., *V*O_2_max, v*V*O_2_max, MMSS, sprint capacity) is needed.

Based on the above, the aim of this systematic review with meta-analysis was to analyze the effect of different ST methods (e.g., HL, SubL, PL, combined training) on running performance (i.e., time trial and time until exhaustion) and its determinants (i.e., *V*O_2_max, v*V*O_2_max, MMSS, sprint capacity) in middle-distance and long-distance runners.

## Methods

The 2020 PRISMA (Preferred Reporting Items for Systematic Reviews and Meta-Analyses) guidelines [25] were followed for this systematic review and meta-analysis. The original protocol was registered on the Open Science Framework before the data analysis (https://osf.io/gyeku).

### Information Sources and Search Strategy

Multiple databases including PubMed, Web of Science (all databases), Scopus, and SPORTDiscus were searched using various search terms and Boolean operators (Table S1 of the Electronic Supplementary Material [ESM]). All articles indexed up to January 2022 were included for selection. The search was updated in November 2022 with notifications of new studies found in the previously searched databases. No restrictions were placed on databases regarding study design, date, language, age, or sex of the participants. Additionally, the reference lists of relevant reviews, systematic reviews, and meta-analyses were reviewed, as well as the reference lists of the articles included in the analysis.

### Selection Process

Two reviewers (LL and SV) reviewed all titles and abstracts obtained from the databases. When the titles and abstracts suggested that the article might meet the inclusion criteria (Table 1), the full article was reviewed. In the case of disagreement between the two reviewers, a third reviewer (RC) was consulted.

**Table 1:** Inclusion and exclusion criteria for meta-analysis

Category	Inclusion criteria	Exclusion criteria
Population	Amateur and competitive middle-distance and long-distance runners (i.e., running distances ≥ 1500 m), aged > 16 years, without restriction to sex or training/competitive level	Subject with injuries, comorbidities, or non-runner endurance athletes
Intervention	A strength training program (i.e., HL, SubL, PL, or a combination of them) which was in addition to, or in partial replacement (i.e., matched training load) of endurance running training, lasting ≥ 3 weeks, with ≥ 1 weekly session	The program includes alternative methods in addition to strength training (e.g., electrical stimulation or body vibration), and/or nutritional supplements (e.g., creatine)
Comparator	Control group that performed endurance running training but did not receive strength training or received it with low loads (< 40% 1RM or > 20RM)	Absence of control group
Outcome	*V*O_2_max, v*V*O_2_max, maximum metabolic steady state, sprint capacity and/or running performance was recorded before and after the strength training intervention	Baseline and/or follow-up data not available
Study design	Randomised and non-randomized controlled studies	Cross-sectional, observational, or case studies

*1RM* one repetition maximum, *HL* high load training, *PL* plyometric training, *RM* repetition maximum, *SubL* submaximal load training, *VO*_*2*_*max* maximal oxygen uptake, *vVO*_*2*_*max* velocity at *V*O_2_max

### Data Collection Process

Data were collected by an independent reviewer (LL), including subject characteristics, methodological data, endurance training, ST intervention, and main outcomes for further analysis. In those articles where only data in the form of figures were presented, the validated WebPlotDigitizer software (Version 4.5; Ankit Rohatgi, Pacifica, CA, USA) [26] was used to extract the data. The reviewers (LL, SV, and RC) discussed the extracted data collectively and discussed any disagreements or controversial data after recoding.

### Eligibility Criteria

Studies were eligible for inclusion according to the PICOS criteria (Participants, Intervention, Comparator, Outcome, and Study design; Table 1).

#### Participants

Subjects over 16 years of age were included in the study, as puberty may influence the adaptive response to training because of hormonal changes that occur during this period [27]. Strength training experience was classified as either experienced or not experienced in ST based on the information provided by each study. The initial *V*O_2_max level was recorded, further categorized by performance level into moderately trained (male individuals ≤ 55 mL/kg/min, female individuals ≤ 45 mL/kg/min), well trained (male individuals 55–65 mL/kg/min, female individuals 45–55 mL/kg/min), or highly trained (≥ 65 mL/kg/min, ≥ 55 mL/kg/min) [28]. When male and female performance levels were not distinguished, ranges were established by averaging the values of both sexes for each respective performance level. If initial *V*O_2_max values were not recorded, performance level was determined according to the participant’s level of competition (moderately trained = recreational or local club level; well trained = collegiate or provincial level; highly trained = national or international level) [19].

#### ST Intervention

Strength training methods were classified according to the training target and training load [18, 19] as follows: (1) HL, defined as programs aiming to improve maximal strength development by performing exercises with high loads (e.g., barbell squat at ≥ 80% 1RM or ≤ 7RM); (2) SubL, defined as programs aiming to improve strength development using exercises with moderate-to-low loads (e.g., maximal power load at 40–79% 1RM or 8–20RM; usually with maximal movement velocity intention); (3) PL, defined as programs aiming to improve stretch–shortening cycle functioning using exercises with light loads or body weight (e.g., jump-based training); and (4) combined training (Combined), defined as programs that included two or more ST methods. The groups that performed ST with very low loads (VL, < 40% 1RM or > 20RM) were considered as a control group. The duration of the intervention was recorded as total weeks, sessions per week, and total number of sessions.

#### Outcome Measurements

Maximal oxygen uptake, v*V*O_2_max, MMSS, sprint capacity, and running performance were recorded before and after the ST interventions. Maximum metabolic steady state was considered if measured as: maximal lactate steady state, second lactate threshold, onset of blood lactate accumulation, lactate turn point, critical speed, or second ventilatory threshold. Sprint capacity was measured as the speed in meters (m/s) or time to cover a distance (s), in efforts where energy resources have been released mainly from glycolysis and phosphates [29]. Running performance was measured by a time trial or time to exhaustion in runs of more than 75 s, where aerobic metabolism predominates [30]. If running performance was measured in more than one test (e.g., 1500 m and 10,000 m), the most similar test between studies was selected. For all outcomes, where the study reported multiple timepoints (i.e., more than two data points), the first record and the last record immediately after the intervention were recorded.

### Risk of Bias, Publication Bias, and Certainty Assessment

The risk of bias of the studies was assessed using the PEDro (Physiotherapy Evidence Database) scale [31, 32], with items 5–7 removed in consideration of the lack of blinding of subjects, assessors, and researchers in supervised exercise interventions [31, 33]. Based on previous criteria [33], the studies were categorized as low risk (≥ 6 points), moderate risk (4–5 points), and high risk (≤ 3 points). To assess the publication bias of the studies on each ST method, a funnel plot was constructed, indicating a publication bias if an asymmetry was observed.

The GRADE (Grading of Recommendations Assessment, Development and Evaluation) approach was used to evaluate the certainty of evidence [34–36]. High certainty of evidence was initially assumed and then downgraded based on the following criteria: risk of bias, downgraded by one or two levels if the median PEDro score was indicative of moderate risk (< 6 points) or high risk (< 4 points), respectively; inconsistency, downgraded by one level if the Cochrane *Q* test for heterogeneity was significant (i.e., *p* < 0.05); indirectness, considered low risk, as the PICOS criteria were ensured; imprecision, downgraded by one level if the number of participants in the control group with the ST group was < 800 or if the confidence interval (CI) was crossed by a small effect size)ES) [i.e., − 0.15 to 0.15]; publication bias, downgraded by one level if an asymmetry was observed in the funnel plot.

### Effect Measures

A standardized mean difference between groups (i.e., control-experimental) was calculated as previously recommended [37]. Effect size was calculated as Hedges’ g corrected for sample size [38] to help deal with small samples [39], which are recurrent in the sport science literature [40]. Where studies reported data as mean and standard error (SE), the standard deviation (SD) was calculated from the SE [41]. The criteria for determining the ES magnitude were established as follows: 0.15, 0.45, and 0.80 for a small, moderate, and large effect, respectively [42].

### Synthesis Methods

A meta-analysis was performed for each ST method (i.e., HL, SubL, PL, or Combined) for each of the main outcomes (i.e., *V*O_2_max, v*V*O_2_max, MMSS, sprint capacity, and running performance) when at least three studies provided an outcome measure [16]. If a study had two or more comparison groups in the same analysis, the sample size of the control group was divided by the number of intervention groups [41]. Because of multiple sources of variation between studies (e.g., training and participant characteristics), a randomized effect model with a restricted maximum likelihood estimation method was conducted for estimating the parameters model (*τ*^2^) recommended over the traditional DerSimonian and Laird method for continuous data [43]. We based the test statistic and CIs in *t*-distribution with a Knapp and Hartung adjustment [44].

To examine heterogeneity between studies, the Cochrane *Q* test was accompanied by the value of *I*^2^ to quantify the effect of heterogeneity, with values of < 25%, 25–75%, and > 75% indicating low, moderate, and high levels of heterogeneity, respectively [41]. Outliers were defined as an ES in which the upper limit of the 95% CI was lower than the lower limit of the pooled effect CI or the lower limit of the 95% CI was higher than the upper limit of the pooled effect CI [45]. A sensitivity analysis was then performed with and without the outlier ES to assess their impact on the analysis [45] (i.e., *p* value from *Q* test).

A moderator analysis was performed to explore factors associated with ES (e.g., subject characteristics; ST intervention characteristics) if at least eight studies were pooled [46, 47], through meta-regression (i.e., age, body mass, height, initial *V*O_2_max, weeks, sessions per week, and total sessions) and sub-group analysis (i.e., sex, performance level, and ST experience). Alpha was set as 0.05. A Comprehensive meta-analysis (Version 3.3.0.70) was used for the analysis and GraphPad Prism 9 (Version 9.2.0) was used to generate the plots.

## Results

### Study Selection

**Fig. 1:**
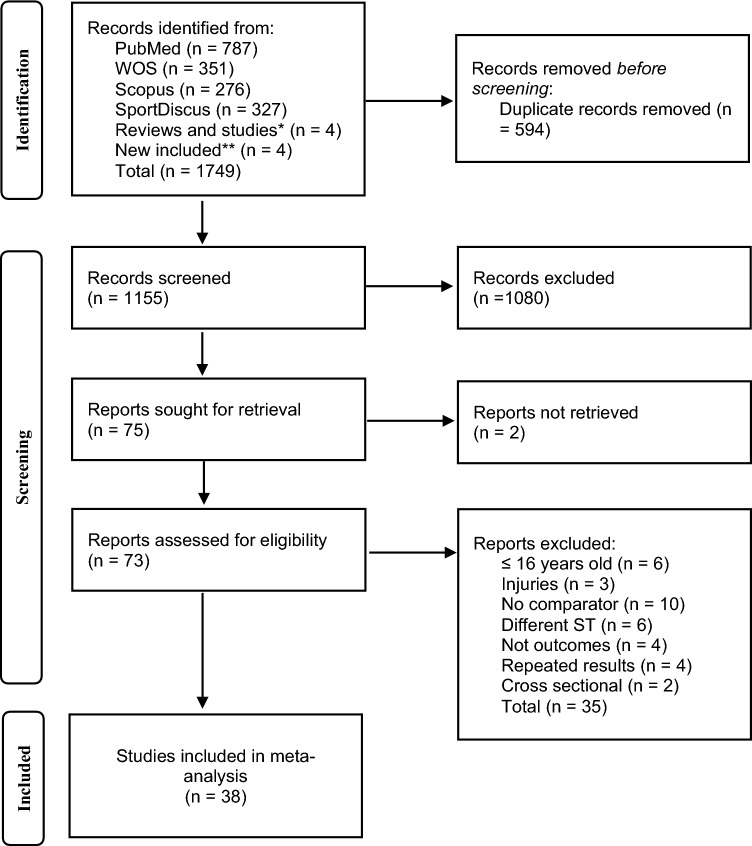
Flow diagram of the studies selection process. *ST* strength training, *WOS* Web of Science, *studies found from notifications of new studies found in the search strategy in the different databases, **studies found in the reference lists of articles, reviews, systematic reviews, and meta-analyses retrieved from our search strategy

The search strategy identified a total of 1749 records (Fig. 1). After removing duplicate records, records not retrieved, and documents excluded after reading the title and/or abstract, 73 studies were assessed for eligibility. Upon full-text reading, 35 studies were excluded because of the following reasons: participants aged under 16 years [48–53] or injured before the intervention [54–56]; no comparator group [57–66]; ST method considered not includable (e.g., core strength training; flywheel and isokinetic eccentric training; local muscular endurance training) [67–72]; no relevant outcomes included (i.e., *V*O_2_max, v*V*O_2_max, MMSS, sprint capacity, running performance) [73–76]; repeated outcome results derived from secondary analysis publications [77–80]; or cross-sectional study [81, 82]. As a result, 38 studies were included in the meta-analyses.

### Study Characteristics

The studies included in the meta-analysis are presented in Table 2 for the characteristics of the participants and the interventions, and in Table 3 for the outcome results included in meta-analyses. Thirty-eight studies were included in at least one analysis: 31 studies measured *V*O_2_max [3, 20–24, 83–107]; 15 studies measured v*V*O_2_max [23, 83–87, 91, 93, 94, 97, 102, 105–108], 21 studies measured MMSS [3, 20, 22–24, 83, 84, 86, 87, 90, 94, 95, 99–101, 105–110], eight studies measured sprint capacity [3, 21, 86, 92, 93, 106, 111, 112], and 24 studies measured running performance [3, 20–23, 85, 89, 90, 92, 95, 97–99, 101, 103, 105, 106, 108–114]. The studies included 894 participants (651 male individuals [290 control and 361 treatments] and 243 female individuals [115 control and 128 treatments]), aged between 17 and 40 years, mean body mass and height of 68.70 kg and 174.50 cm, respectively. Participants were moderately trained (*n* = 324), well trained (*n* = 272), and highly trained (*n* = 298). The ST programs lasted between 6 and 40 weeks, with one to four sessions per week.

**Table 2:** Participant and strength training intervention characteristics of the included studies

Study	G	Subject characteristics							Strength training intervention						
		*n*	Age, years	BM	H	VO_2_max	PLv	ST Exp	D	F	TS	Exercises	Load	Set × repetition	Rest
Ache-Dias et al*.* [83]	PL	9 (M = 4; F = 5)	24	63	160	50	MT	NR	8	2	16	PL: continuous jump	BW	4–6 × 30 s	5 min
	C	9 (M = 4; F = 5)	31	66	170	49	MT	NR							
Bachero-Mena et al. [111]	SubL + PL	6 (M)	22	65	179	NR	HT	Yes	25	2	50	SubL: squat. PL: jump squat, step phase of triple jump, resisted sprint	SubL: 40–55% 1RM. PL: low load: BW	SubL: 2–3 × 4–6. PL: 3–5 × 4–16	–
	C	7 (M)	23	62	172	NR	HT	Yes	25	2	50	VL: half squat, step-up, hamstrings, quadriceps, calf raises, jump squat, jump	0–40% 1RM	3–5 × 10–30	–
Beattie et al. [84]	HL + SubL + PL	11 (M)	30	73	183	60	WT	No	40	1.5	60	HL: back squat. SubL: single-leg squat, reverse lung, Romanian deadlift, single-leg Romanian deadlift, single jump, split squat, skater squat. PL: pogo jump, countermovement jump, continuous countermovement jump, drop jump	HL: high load. SubL: medium to high load. PL: low load	HL: 2–5 × 1–12. SubL: 1–3 × 3. PL: 2–3 × 4–6	–
	C	9 (M)	27	70	177	63	WT	No							
Berryman et al. [85]	SubL	12 (M)	31	76	176	58	WT	No	8	1	8	SubL: half squat	Power peak load	3–6 × 8	3 min
	PL	11 (M)	29	75	178	58	WT	No	8	1	8	PL: drop jump (20–60 cm)	BW	3–6 × 8	3 min
	C	5 (M)	29	76	180	56	WT	No							
Bertuzzi et al. [22]	HL	8 (M)	31	70	174	59	WT	No	6	2	12	HL: half squat	2 wk: 75–80% 1RM; 2 wk: 80–85% 1RM; 2 wk: 85–90% 1RM	3–6 × 4–10	3 min
	C	6 (M)	33	69	173	58	WT	No							
Blagrove et al. [19]	SubL + PL	9 (M = 4; F = 5)	17	58	170	59	HT	No	10	2	20	PL: box jump, a-skip, hurdle jump and land, single-leg box jump, high-knees, depth jump. SubL: back squat, Romanian deadlift, single-leg press, calf raises, rack pull, deadlift, step-up	PL: BW. SubL: moderate load	PL: 3–4 × 6–8. SubL: 2–3 × 6–12	PL: 1.5 min. SubL: 3 min
	C	9 (M = 4; F = 5)	18	59	172	62	HT	No							
Damasceno et al*.* [87]	HL	9 (M)	34	68	174	54	MT	NR	8	2	16	HL: half squat, leg press, plantar flexion, knee extension	2 wk: 75–80% 1RM; 2 wk: 80–85% 1RM; 2 wk: 85–90% 1RM; 2 wk: 87–93% 1RM	2–3 × 3–10	3 min
	C	9 (M)	33	71	174	56	WT	NR							
do Carmo et al. [23]	PL	15 (M)	33	70	172	55	WT	No	8	2	16	PL: squat jump, split scissor jump, double-leg bound, alternate leg bound, single-leg forward hop, depth jump, double-leg hurdle jump, single-leg hurdle hop	BW	2–5 × 6	-
	C	13 (M)	33	70	172	57	WT	No							
Ferrauti et al. [24]	HL	11 (M = 9; F = 2)	40	77	NR	52	WT	No	8	2	16	HL: leg press, knee extension, knee flexion, hip extension, ankle extension	87–93% 1RM	4 × 3–5	3 min
	C	11 (M = 7; F = 4)	40	70	NR	51	WT	No							
Filipas et al*.* [20]	PL	15 (M)	34	64	NR	69	HT	No	7	1	7	PL: vertical jump, horizontal jump, bounce drop jump	BW	2 × 10	2 min
	C	15 (M)	34	65	NR	68	HT	No							
	PL	15 (M)	35	65	NR	69	HT	No	7	1	7	PL: vertical jump, horizontal jump, bounce drop jump	BW	2 × 10	2 min
	C	15 (M)	34	65	NR	68	HT	No							
García-Pinillos et al. [113]	PL	44 (M = 23; F = 21)	27	66	172	NR	MT	No	10	3	30	PL: jump-rope bilateral, unilateral, unilateral-alternating	BW	5 min	30 s works: 30 s rest
	C	42 (M = 18; F = 24)	26	66	171	NR	MT	No							
Hamilton et al. [108]	PL	10 (M)	28	72	178	66	HT	NR	6	1.5	9	PL: single-leg step-up, jump	BW	3 × 20	2 min
	C	10 (M)	31	73	178	66	HT	NR							
Johnston et al. [88]	HL + SubL	6 (F)	30	57	163	51	WT	No	10	3	30	SubL: bent-leg, heel raise, straight-leg heel raise. HL: knee flexion, knee extension, squat, hammer curl, lunge	SubL: 60–67% 1RM. HL: 80–85% 1RM	SubL: 2 × 12–20. HL: 3 × 6–8	2 min
	C	6 (F)	30	52	164	52	WT	No							
Karsten et al. [109]	HL	8 (M = 5; F = 3)	39	74	177	47	MT	NR	6	2	12	HL: Romanian deadlift, squat, calf raises, lunges	80% 1RM	4 × 4	2 min
	C	8 (M = 6; F = 2)	30	69	175	47	MT	NR							
Kelly et al. [89]	HL	7 (F)	21	66	162	40	MT	No	10	3	30	HL: squat, calf raises, hip extension, hip flexion, hamstrings curl	1 wk: 60–70% 1RM; 1 wk: 70–80% 1RM; 8 wk: ≥ 85% 1RM	3 × 5	3–4 min
	C	9 (F)	20	60	166	40	MT	No							
Li et al. [21]	HL + PL	10 (M)	20	58	178	66	HT	NR	8	3	24	HL: back squat, Bulgarian squat, Romanian deadlifts. PL: double-leg hurdle hop, single-leg hop, drop jump	HL: 80–85% 1RM. PL: BW	HL: 3 × 5 PL: 3 × 6	4 min
	HL	9 (M)	21	58	175	66	HT	NR	8	3	24	HL: back squat, Bulgarian squat, Romanian deadlift	80–85% 1RM	5 × 5	3 min
	C	9 (M)	21	61	179	66	HT	NR	8	3	24	VL: back squat, Bulgarian squat, Romanian deadlift	40% 1RM	5 × 20–30	1 min
Lum et al. [90]	PL	7 (M)	29	66	171	54	MT	NR	6	2	12	PL: alternate leg bounding, double-leg and single-leg hurdle hop, depth jump	BW	3–4 × 5–40	3 min
	C	7 (M)	29	66	171	54	MT	NR							
Lum et al. [91]	PL	9 (M = 6; F = 3)	38	65	170	51	WT	Yes	6	2	12	PL: depth jump, single-leg bounding, split jump	BW	2–4 × 4–5	3 min
	C	8 (M = 6; F = 2)	32	62	168	50	WT	Yes	6	2	12	VL: squat, lunge with knee lift, arabesque	BW	3 × 30 s	30 s
Lundstrom et al*.* [92]	PL	11 (M = 7; F = 4)	21	66	NR	51	WT	NR	12	1	12	PL: horizontal jump, vertical jump, frog hop, alternate leg bound, single-leg forward hop, lateral cone jump, forward/backward cone jump, squat jump, lunge jump, depth jump, box jump	BW	1–2 × 8–20	1 min
	C	11 (M = 6; F = 5)	20	74	NR	54	WT	NR	12	1	12	Core exercises	BW	1–3 × 10–30	1 min
Machado et al. [114]	PL	8 (M)	38	81	177	NR	MT	NR	8	2	16	PL: multi-direction jumps, squat jump	BW	6 × 30 s	30 s
	PL	8 (M)	39	84	179	NR	MT	NR	8	2	16	PL: multi-direction jumps, drop jump	BW	6 × 30 s	30 s
	C	8 (M)	35	79	176	NR	MT	NR							
Mikkola et al. [93]	SubL + PL	13 (M = 9; F = 4)	17	63	177	62	HT	NR	8	3	24	PL: alternate jump, calf jump, squat jump, hurdle jump. SubL: half squats, knee extensions, knee flexions, calf raises	Low-load or BW	2–3 × 6–10	–
	C	12 (M = 9; F = 3)	17	60	172	62	HT	NR							
Mikkola et al. [107]	HL	11 (M)	36	78	179	51	MT	Yes	8	2	16	HL: squat, seated leg press	85–90% 1RM	3 × 4–6	2–3 min
	SubL + PL	10 (M)	36	79	181	51	MT	Yes	8	2	16	SubL: squat, seated leg press. PL: squat jump, scissor jump	0–40% 1RM	SubL: 3 × 6. PL: 2 × 5–10	2–3 min
	C	6 (M)	34	84	181	48	MT	Yes	8	2	16	VL: squat, lunges	BW	3 × 40–50 s	10–20 s
Millet et al. [94]	HL	7 (M)	24	67	175	70	HT	NR	14	2	28	HL: hamstrings curl, leg press, seated press, squat, leg extension, heel raises	87–93% 1RM	3–5 × 3–5	–
	C	8 (M)	21	65	175	68	HT	NR							
Paavolainen et al*.* [3]	PL + SubL	10 (M)	23	72	179	68	HT	NR	9	2.5	22.5	PL: alternate jump, bilateral countermovement jump, drop jump, hurdle jump, one-legged. SubL: leg press, knee extensor-flexor	PL: BW or barbell. SubL: 0–40% 1RM	–	–
	C	8 (M)	24	70	182	68	HT	NR	–	–	–	VL: leg exercises	BW	1 × 12	–
Pellegrino et al. [95]	PL	11 (M = 7; F = 4)	33	68	172	48	MT	No	6	2.5	15	PL: squat jump, split scissor jump, double-leg bound, alternate leg bound, single-leg forward hop, deep knee bend box jump landing, double-leg hurdle jump, single-leg hurdle hop	BW	2–3 × 8–15	–
	C	11 (M = 7; F = 4)	34	71	171	48	MT	No							
Ramírez-Campillo et al. [112]	PL	17 (M = 9; F = 8)	22	60	NR	NR	HT	No	6	2	12	PL: vertical jump, horizontal jump, drop jump	BW	2 × 10	2 min
	C	15 (M = 10; F = 5)	22	60	NR	NR	HT	No							
Saunders et al. [96]	PL	7 (M)	23	68	NR	68	HT	Yes	9	2.5	26	PL: countermovement jump, knee lift, ankle jump, hamstrings curls, alternate leg bound, skip for height, single-leg ankle jump, continuous hurdle jump, scissor jump for height. SubL: leg press^a^	60% 1RM + BW	1–5 × 6–20	–
	C	8 (M)	25	68	NR	70	HT	Yes							
Schumann et al. [110]	HL + SubL + PL	13 (M)	33	78	179	NR	MT	NR	24	2	46	HL: leg press, single and double knee flexion, calf raises. SubL: squat jump, drop jump, leaps, step-up. PL: squat jump, drop jump, leap	4 wk: 40–50% 1RM; 20 wk: 60–85% 1RM	SubL HL: 5–12 sets	1–3 min
	C	14 (M)	33	78	179	NR	MT	NR							
Sedano et al. [97]	SubL + PL	6 (M)	24	69	181	69	HT	Yes	12	2	24	SubL: barbell squat, lying leg curl, seated calf raises, leg extension. PL: vertical jump over hurdles (40 cm), horizontal jump	SubL: 70% 1RM. PL: BW	SubL: 3 × 7. PL: 10	5 min
	SubL	6 (M)	24	66	179	71	HT	Yes	12	2	24	SubL: barbell squat, lying leg curl, seated calf raises, leg extension	40% 1RM	3 × 20	1 min
	C	6 (M)	24	70	185	69	HT	Yes	12	2	24	VL: squat with band, lying leg curl with band, calf raises with band, leg extension with band	Resistance band	1 × 25	5 min
Skovgaard et al*.* [98]	HL	12 (M)	31	77	180	61	WT	No	8	4	32	HL: squat, deadlift, leg press	2 wk 65–67% 1RM; 6 wk: HL: 80–90% 1RM	HL: 3 × 8–4	3 min
	C	9 (M)	31	77	180	59	WT	No							
Spurrs et al. [99]	PL	8 (M)	25	74.7	178	58	WT	No	6	2.5	15	PL: squat jump, split scissor jump, double-leg bound, alternate leg bound, single-leg forward hop, depth jump, double-leg hurdle jump, single-leg hurdle hop	BW	2–3 × 8–15	–
	C	9 (M)	25	70.2	178	58	WT	No							
Štohanzl et al. [100]	PL	9 (F)	32	69	169	39	MT	No	10	2	20	PL: calf jump, low skater jump, half squat jump	BW	3–4 × 12–3	2 min
	PL	11 (F)	32	71	169	34	MT	No	10	1	10	PL: calf jump, low skater jump, half squat jump	BW	3–4 × 12–3	2 min
	C	11 (F)	32	69	169	37	MT	No							
Støren et al. [101]	HL	8 (M = 4; F = 4)	29	60	171	61	HT	No	8	3	24	HL: half squat	90% 1RM	4 × 4	3 min
	C	9 (M = 5; F = 4)	30	71	179	57	WT	No							
Taipale et al. [102]	HL + PL	9 (F)	29	62	168	45	MT	NR	16	1.2	24	HL: squats, leg press. PL: box jump, vertical jump	HL: 85–90% 1RM. PL: BW	HL: 2–3 × 4–6. PL: 2–3 × 8–10	2–3 min
	C	9 (F)	35	60	165	43	MT	NR	16	1.2	24	VL: squats, lunges, sit-up, calf-raises, step-up	BW	1 × 45–50 s	15–20 s
	HL + PL	9 (M)	31	78	178	50	MT	NR	16	1.2	24	HL: Squat, leg press. PL: box jump, vertical jump	HL: 85–90% 1RM. PL: BW	HL: 2–3 × 4–6. PL: 2–3 × 8–10	2–3 min
	C	7 (M)	34	84	180	46	MT	NR	16	1.2	24	VL: squats, lunges, sit-up, calf-raises, step-up	BW	1 × 45–50 s	15–20 s
Trowell et al*.* [103]	SubL + PL	14 (M = 8; F = 6)	33	73	173	61	HT	No	10	2	20	PL: ankle bouncing, hurdle jump (40 cm), high-knee drill or a-skip drill, split squat jump, countermovement jump or drop jump (45 cm). SubL: back squat, single-leg deadlift	SubL: 70% 1RM. PL: 0–30% BW	SubL: 2–5 × 10. PL: 2–5 × many	3 min
	C	14 (M = 9; F = 5)	34	70	175	66	HT	No	10	2	20	VL: sit-up, lunge, among others	BW	–	–
Turner et al. [104]	PL	10 (M = 4; F = 6)	31	65	170	50	WT	NR	6	3	18	PL: vertical jump, one-legged vertical jump, vertical springing jump, split-squat jump, incline jump	BW	1 × 5–15	–
	C	8 (M = 4; F = 4)	27	72	174	54	WT	NR							
Vikmoen et al*.* [105]	HL	11 (F)	35	62	169	52	WT	No	11	2	22	HL: half squat, one-legged leg press, standing one-legged hip flexion, ankle plantar flexion	3 wk: 75–85% 1RM; 3 wk: 80–87% 1RM; 5 wk: 85–90% 1RM	3 × 4–10	–
	C	8 (F)	32	66	170	54	WT	No							
Vorup et al. [106]	HL	9 (M)	39	75	181	60	WT	NR	8	2	16	HL: half squat, leg press, deadlift	1 wk: 75% 1RM; 1 wk: 80% 1RM; 2 wk: 85% 1RM; 4 wk: 90% 1RM	1–4 × 10–4	3 min
	C	7 (M)	37	74	184	60	WT	NR							

*1RM* one repetition maximum, *BM* body mass (kg), *C* control, *D* duration (weeks), *F* frequency (session/week), *F* female, *G* group, *H* height (cm), *HL* high load training, *HT* highly trained, *M* male; *min* minutes, *MT* moderately trained, *n* sample size, *PL* plyometric training, *PLv* performance level, *s* seconds, *SubL* submaximal load training, *ST exp* strength training experience, *TS* total sessions, *VL* very low load, *VO*_*2*_*max* (mL/kg/min), *WL* well trained, *wk* weeks

^a^The exercise was not considered relevant to be included as a strength training method in this group

**Table 3:** Analysis of the studies included in the meta-analysis of *V*O_2_max, v*V*O_2_max, maximum metabolic steady state, sprint capacity, and running performance

Study	Group	*n*	*V*O_2_max		v*V*O_2_max		Maximum metabolic steady state			Sprint capacity			Running performance		
			Mean pre (SD)	Mean post (SD)	Mean pre (SD)	Mean post (SD)	Measurement	Mean pre (SD)	Mean post (SD)	Test	Mean pre (SD)	Mean post (SD)	Test	Mean pre (SD)	Mean post (SD)
Ache-Dias et al. [83]	PL	9 (M = 4; F = 5)	3.17 (0.72)	3.46 (0.70)	13.68 (1.40)	14.06 (1.57)	sOBLA	10.11 (1.64)	11.09 (1.82)						
	C	9 (M = 4; F = 5)	3.23 (0.73)	3.25 (0.72)	14.00 (1.33)	14.00 (1.26)	sOBLA	11.39 (1.8)	11.46 (1.77)						
Bachero-Mena et al. [111]	SubL + PL	6 (M)								20-m tt (s)	2.91 (0.12)	2.87 (0.08)	800 m tt (s)	112.79 (6.42)	111.35 (5.79)
	C	7 (M)								20-m tt (s)	2.95 (0.08)	2.9 (0.06)	800 m tt (s)	117.97 (1.97)	116.11 (1.17)
Beattie et al. [84]	HL + SubL + PL	11(M)	59.60 (2.50)	61.60 (5.20)	20.15 (0.91)	20.95 (0.96)	s4 mmol/L Bla	16.46 (1.20)	16.81 (1.30)						
	C	9 (M)	63.20 (2.90)	65.00 (3.20)	21.17 (1.03)	21.5 (1.03)	s4 mmol/L Bla	17.10 (1.04)	17.49 (0.93)						
Berryman et al. [85]	SubL	12 (M)	57.50 (6.70)	56.10 (6.70)	16.60 (1.50)	17.30 (1.60)							3000-m tt (s)	755.00 (87.00)	724.00 (77.00)
	PL	11 (M)	57.50 (6.50)	57.30 (5.50)	16.60 (1.30)	17.30 (1.30)							3000-m tt (s)	748.00 (81.00)	712.00 (76.00)
	C	5 (M)	55.70 (8.20)	55.30 (8.90)	17.30 (2.00)	17.50 (2.40)							3000-m tt (s)	711.00 (107.00)	690.00 (109.00)
Bertuzzi et al. [22]	HL	8 (M)	58.50 (7.60)	58.20 (6.20)	19.00 (1.00)	19.00 (1.00)	RCP (%v*V*O_2_max)	85.00 (7.00)	86.00 (8.00)				Time to exhaustion at v*V*O_2_max	454.90 (228.00)	562.20 (255.00)
	C	6 (M)	57.60 (6.30)	57.50 (5.40)	18.00 (2.00)	18.00 (2.00)	RCP (%v*V*O_2_max)	86.00 (6.00)	85.00 (6.00)				Time to exhaustion at v*V*O_2_max	305.10 (201.60)	315.30 (184.00)
Blagrove et al. [19]	SubL + PL	9 (M = 4; F = 5)	229.20 (41.30)	227.50 (36.20)	16.80 (2.40)	17.30 (2.60)	s4 mmol/L Bla	14.90 (2.40)	15.40 (2.50)	20-m tt (s)	2.73 (0.22)	2.69 (0.19)			
	C	9 (M = 4; F = 5)	241.20 (24.20)	242.00 (21.50)	17.80 (0.80)	17.80 (1.70)	s4 mmol/L Bla	15.80 (1.00)	16.40 (1.40)	20-m tt (s)	2.64 (0.24)	2.62 (0.23)			
Damasceno et al. [87]	HL	9 (M)	54.30 (5.40)	54.40 (5.30)	16.70 (1.30)	17.20 (1.60)	RCP (km/h)	15.40 (1.10)	16.00 (1.20)						
	C	9 (M)	55.80 (5.30)	56.80 (6.00)	17.60 (1.10)	17.70 (1.50)	RCP (km/h)	15.80 (1.00)	15.70 (1.20)						
do Carmo et al. [23]	PL	15 (M)	55.10 (4.30)	56.90 (4.70)	18.20 (0.80)	18.50 (0.80)	VT_2_ (mL/kg/min)	49.90 (3.80)	51.60 (4.20)				10,000-m tt (s)	2508.00 (154.00)	2482.00 (157.00)
	C	13 (M)	56.70 (6.10)	57.20 (6.70)	18.70 (1.30)	18.70 (1.60)	VT_2_ (mL/kg/min)	50.00 (5.80)	50.90 (5.60)				10,000-m tt (s)	2445.00 (219.00)	2458.00 (215.00)
Ferrauti et al. [24]	HL	11 (M = 9; F = 2)	52.00 (6.10)	54.90 (4.40)			s4 mmol/L Bla	3.54 (0.41)	3.69 (0.46)						
	C	11 (M = 7; F = 4)	51.10 (7.50)	51.10 (7.50)			s4 mmol/L Bla	3.40 (0.51)	3.49 (0.52)						
Filipas et al. [20]	PL	15 (M)	68.53 (3.00)	69.00 (3.10)			s4 mmol/L Bla	17.28 (0.50)	17.70 (0.50)				5000-m tt (s)	994.90 (29.00)	978.00 (29.00)
	C	15 (M)	67.30 (2.80)	68.00 (2.50)			s4 mmol/L Bla	17.40 (0.63)	17.50 (0.70)				5000-m tt (s)	993.00 (31.90)	984.00 (33.00)
	PL	15 (M)	68.90 (2.90)	69.90 (3.70)			s4 mmol/L Bla	17.31 (0.60)	17.70 (0.50)				5000-m tt (s)	992.90 (27.00)	975.00 (24.00)
	C	15 (M)	68.20 (3.10)	68.30 (4.10)			s4 mmol/L Bla	17.30 (0.72)	17.50 (0.80)				5000-m tt (s)	998.00 (34.50)	990.00 (37.00)
García-Pinillos et al. [113]	PL	44 (M = 23; F = 21)											3000-m tt (s)	774.60 (79.50)	751.70 (65.80)
	C	42 (M = 18; F = 24)											3000-m tt (s)	762.10 (87.50)	750.80 (83.60)
Hamilton et al. [108]	PL	10 (M)			20.50 (1.30)	21.05 (1.32)	s4 mmol/L Bla	14.70 (2.00)	15.29 (2.05)				5000-m tt (s)	1128.00 (78.00)	1103.40 (66.60)
	C	10 (M)			20.40 (1.00)	20.58 (1.02)	s4 mmol/L Bla	15.50 (1.30)	15.58 (1.37)				5000-m tt (s)	1098.00 (78.00)	1087.00 (63.00)
Johnston et al. [88]	HL + SubL	6 (F)	50.50 (2.20)	48.00 (2.00)											
	C	6 (F)	51.50 (2.40)	51.00 (1.90)											
Karsten et al. [109]	HL	8 (M = 5; F = 3)					CS (km/h)	13.80 (2.10)	14.20 (1.70)				5000-m tt (s)	1288.30 (183.40)	1242.90 (187.00)
	C	8 (M = 6; F = 2)					CS (km/h)	14.7 (1.80)	14.7 (1.90)				5000-m tt (s)	1264.40 (203.70)	1270.30 (221.80)
Kelly et al. [89]	HL	7 (F)	39.90 (5.20)	45.10 (7.20)									3000-m tt (s)	957.00 (93.00)	847.00 (48.00)
	C	9 (F)	39.50 (6.00)	42.30 (4.90)									3000-m tt (s)	1010.00 (150.00)	933.00 (107.00)
Li et al*.* [21]	HL + PL	10 (M)	65.65 (5.06)	64.47 (4.31)						50-m tt (s)	6.25 (0.19)	6.11 (0.24)	5000-m tt (s)	953.70 (12.30)	926.90 (9.92)
	HL	9 (M)	65.54 (5.52)	64.65 (6.18)						50-m tt (s)	6.17 (0.33)	6.04 (0.33)	5000-m tt (s)	952.56 (10.10)	932.67 (11.61)
	C	9 (M)	66.14 (5.25)	67.79 (3.03)						50-m tt (s)	5.94 (0.21)	5.92 (0.30)	5000-m tt (s)	954.11 (6.75)	947.33 (10.03)
Lum et al. [90]	PL	7 (M)	54.40 (5.00)	53.70 (6.70)			sLT_2_	12.40 (1.00)	12.40 (1.00)				10,000-m tt (s)	3028.00 (405.00)	2915.00 (420.00)
	C	7 (M)	53.90 (7.40)	54.60 (6.70)			sLT_2_	12.20 (1.50)	12.30 (1.50)				10,000-m tt (s)	3181.00 (481.00)	3061.00 (421.00)
Lum et al. [91]	PL	9 (M = 6; F = 3)	50.60 (5.20)	52.60 (6.30)	16.10 (1.60)	16.80 (1.80)							2400-m tt (s)	598.00 (61.00)	584.00 (64.00)
	C	8 (M = 6; F = 2)	49.90 (5.30)	50.30 (5.70)	15.60 (1.70)	15.70 (1.80)							2400-m tt (s)	616.00 (46.00)	614.00 (45.00)
Lundstrom et al. [92]	PL	11 (M = 7; F = 4)	50.50 (8.80)	57.80 (8.30)						60-m tt (s)	10.45 (1.37)	9.88 (1.51)	2-mile tt (s)	882.00 (144.00)	720.00 (138.00)
	C	11 (M = 6; F = 5)	53.60 (8.30)	57.40 (7.50)						60-m tt (s)	9.83 (1.4)	9.67 (1.19)	2-mile tt (s)	840.00 (120.00)	798.00 (102.00)
Machado et al. [114]	PL	8 (M)											5000-m tt (s)	1495.50 (227.98)	1355.00 (134.67)
	PL	8 (M)											5000-m tt (s)	1470.25 (169.24)	1299.63 (191.47)
	C	8 (M)											5000-m tt (s)	1701.00 (130.09)	1696.25 (139.35)
Mikkola et al. [93]	SubL + PL	13 (M = 9; F = 4)	62.40 (5.40)	62.80 (5.80)	17.20 (1.00)	17.40 (1.00)				30 m (m/s)	8.08 (0.16)	8.17 (0.17)			
	C	12 (M = 9; F = 3)	61.80 (5.30)	62.80 (5.60)	17.10 (1.00)	17.30 (1.40)				30 m (m/s)	7.88 (0.18)	7.85 (0.17)			
Mikkola et al. [107]	HL	11 (M)	51.00 (4.00)	52.00 (5.00)	15.10 (1.20)	15.30 (1.10)	sMMSS	12.70 (1.20)	13.20 (1.20)						
	SubL + PL	10 (M)	51.00 (5.00)	52.00 (4.00)	15.30 (1.00)	15.60 (1.00)	sMMSS	12.60 (0.80)	13.00 (1.00)						
	C	6 (M)	48.00 (6.00)	52.00 (6.00)	14.50 (0.90)	15.20 (1.00)	sMMSS	11.80 (0.70)	12.40 (0.80)						
Millet et al. [94]	HL	7 (M)	69.70 (3.60)	67.20 (4.40)	19.52 (0.94)	19.99 (0.79)	VT_2_ (%*V*O_2_max)	88.40 (2.80)	88.10 (5.00)						
	C	8 (M)	67.60 (6.40)	67.30 (5.60)	19.32 (0.94)	19.80 (1.18)	VT_2_ (%*V*O_2_max)	89.30 (8.10)	88.80 (6.40)						
Paavolainen et al. [3]	PL + SubL	10 (M)	67.70 (2.80)	70.20 (3.20)			LT_2_ (mL/kg/min)	47.30 (3.30)	48.10 (3.50)	20 m (m/s)	7.96 (0.57)	8.23 (0.54)	5000-m tt (s)	1103.40 (21.60)	1066.80 (6.00)
	C	8 (M)	68.30 (3.10)	68.40 (3.60)			LT_2_ (mL/kg/min)	48.90 (4.50)	49.30 (2.80)	20 m (m/s)	8.28 (0.35)	8.08 (0.31)	5000-m tt (s)	1073.40 (24.00)	1084.80 (35.40)
Pellegrino et al. [95]	PL	11 (M = 7; F = 4)	48.00 (1.80)	50.50 (2.10)			sOBLA	13.79 (0.50)	13.97 (0.50)				3000-m tt (s)	780.90 (29.90)	760.80 (29.10)
	C	11 (M = 7; F = 4)	47.70 (2.30)	49.20 (2.10)			sOBLA	12.53 (0.43)	12.60 (0.50)				3000-m tt (s)	830.40 (35.60)	817.20 (39.80)
Ramírez-Campillo et al. [112]	PL	17 (M = 9; F = 8)								20-m tt (s)	3.92 (0.30)	3.83 (0.30)	2400-m tt (s)	456.00 (42.00)	438.00 (48.00)
	C	15 (M = 10; F = 5)								20-m tt (s)	3.97 (0.20)	3.94 (0.40)	2400-m tt (s)	480.00 (54.00)	474.00 (54.00)
Saunders et al*.* [96]	PL	7 (M)	67.70 (6.20)	68.20 (6.20)											
	C	8 (M)	70.4 (6.20)	72.5 (5.00)											
Schumann et al. [110]	HL + SubL + PL	13 (M)					s4 mmol/L BLa	3.90 (0.40)	4.10 (0.40)				1000-m tt (s)	228.00 (48.00)	198.00 (12.00)
	C	14 (M)					s4 mmol/L BLa	3.70 (0.50)	3.90 (0.30)				1000-m tt (s)	216.00 (24.00)	198.00 (12.00)
Sedano et al. [97]	SubL + PL	6 (M)	68.83 (1.94)	69.51 (1.98)	20.91 (0.90)	21.70 (0.78)							3000-m tt (s)	491.85 (1.93)	488.65 (1.87)
	SubL	6 (M)	70.73 (2.88)	71.45 (1.76)	21.45 (1.67)	22.45 (1.69)							3000-m tt (s)	492.63 (1.35)	491.79 (1.02)
	C	6 (M)	68.80 (1.83)	69.20 (2.05)	21.95 (1.21)	22.12 (1.02)							3000-m tt (s)	493.30 (1.24)	493.51 (1.52)
Skovgaard et al. [98]	HL	12 (M)	60.70 (1.20)	59.50 (0.80)									1500-m tt (s)	327.00 (8.00)	310.00 (5.00)
	C	9 (M)	58.90 (2.10)	58.20 (2.30)									1500-m tt (s)	322.00 (6.00)	327.00 (9.00)
Spurrs et al. [99]	PL	8 (M)	57.60 (7.70)	59.50 (8.10)			LT_2_ (mmol/L BLa)	4.26 (1.18)	4.03 (1.42)				3000-m tt (s)	616.80 (75.60)	607.20 (69.00)
	C	9 (M)	57.80 (5.40)	61.50 (5.90)			LT_2_ (mmol/L BLa)	3.73 (1.08)	4.10 (0.73)				3000-m tt (s)	561.60 (34.20)	558.60 (31.20)
Štohanzl et al. [100]	PL	9 (F)	38.80 (1.70)	39.70 (3.00)			VT_2_ (mL/kg/min)	28.90 (2.70)	28.40 (2.60)						
	PL	11 (F)	33.70 (6.40)	34.20 (6.80)			VT_2_ (mL/kg/min)	31.20 (3.50)	30.80 (3.50)						
	C	11 (F)	37.30 (5.10)	38.30 (4.50)			VT_2_ (mL/kg/min)	30.20 (3.40)	31.60 (3.80)						
Støren et al. [101]	HL	8 (M = 4; F = 4)	61.40 (5.10)	61.00 (5.80)			LT_2_ (%*V*O_2_max)	83.00 (4.00)	83.00 (6.00)				Time to exhaustion at v*V*O_2_max	337.00 (124.00)	409.00 (164.00)
	C	9 (M = 5; F = 4)	56.50 (8.20)	56.00 (7.00)			LT_2_ (%*V*O_2_max)	85.00 (4.00)	87.00 (5.00)				Time to exhaustion at v*V*O_2_max	412.00 (163.00)	371.00 (134.00)
Taipale et al. [102]	HL + PL	9 (F)	43.70 (2.40)	45.40 (2.70)	12.83 (0.83)	13.45 (0.79)									
	C	9 (F)	42.80 (5.90)	44.80 (6.40)	12.92 (1.58)	13.31 (1.36)									
	HL + PL	9 (M)	50.90 (5.40)	51.70 (5.40)	14.80 (1.18)	15.55 (1.10)									
	C	7 (M)	45.70 (3.00)	49.800 (7.00)	13.93 (0.75)	15.02 (1.05)									
Trowell et al. [103]	SubL + PL	14 (M = 8; F = 6)	60.56 (3.77)	60.56 (4.33)									2000-m tt (s)	494.22 (41.24)	480.49 (41.78)
	C	14 (M = 9; F = 5)	65.60 (7.49)	64.16 (7.10)									2000-m tt (s)	482.86 (62.64)	480.68 (61.88)
Turner et al. [104]	PL	10 (M = 4; F = 6)	50.40 (9.00)	50.40 (8.00)											
	C	8 (M = 4; F = 4)	54.00 (7.20)	54.20 (6.40)											
Vikmoen et al. [105]	HL	11 (F)	52.20 (2.30)	52.70 (3.30)	12.80 (0.70)	13.00 (0.90)	s3.5 mmol/L BLa	9.59 (0.68)	9.69 (0.64)				40-min all-out (km)	6.35 (0.30)	6.45 (0.38)
	C	8 (F)	54.20 (2.90)	53.10 (1.90)	13.10 (0.50)	13.30 (0.60)	s3.5 mmol/L BLa	9.82 (0.50)	10.03 (0.54)				40-min all-out (km)	6.50 (0.19)	6.66 (0.23)
Vorup et al. [106]	HL	9 (M)	59.90 (5.70)	59.80 (5.60)	18.60 (0.40)	18.00 (0.40)	LT_2_ (mmol/L BLa)	5.70 (0.70)	5.50 (0.50)	400 m tt (s)	66.05 (5.02)	62.82 (4.26)	10,000-m tt (s)	2407.20 (148.92)	2363.82 (155.82)
	C	7 (M)	60.20 (5.90)	60.10 (5.50)	18.10 (1.30)	18.30 (1.20)	LT_2_ (mmol/L BLa)	5.70 (1.00)	5.60 (1.10)	400 m tt (s)	63.82 (5.16)	63.58 (5.60)	10,000 m tt (s)	2424.60 (160.74)	2400.30 (203.16)

*BLa* blood lactate, *C* control, *CS* critical speed (km/h), *F* female, *HL* high load training, *LT*_*2*_ second lactate threshold, *M* male, *min* minute, *n* sample size, *PL* plyometric training, *RCP* respiratory compensation point, *SD* standard deviation, *SubL* submaximal load training, *s* seconds, *s3.5 mmol/L BLa* speed at 3.5 mmol/L BLa (km/h), *s4 mmol/L BLa* speed at 4 mmol/L BLa (km/h), *sMMSS* speed at maximal metabolic steady state (km/h), *sLT*_*2*_ speed at second lactate threshold (km/h), *sOBLA* speed at onset of blood lactate accumulation, *tt* time trial, *VO*_*2*_*max* (mL∙kg^−1^∙min^−1^), *vVO*_*2*_*max* (km/h), *VT*_*2*_ second ventilatory threshold

### Risk of Bias, Publication Bias, and Certainty Assessment

The median of risk of bias was 6 (range from 4 to 7; moderate-to-low risk of bias; Table S2 of the ESM). Publication bias was found only in the analysis of running performance in the combined group (Fig. S1 of the ESM). The results of the certainty of the evidence for each outcome are presented in Table 4. The reasons for downgrading by one or more levels of certainty were (1) risk (moderate) of bias, (2) inconsistency (i.e., significant heterogeneity was found), (3) imprecision (i.e., low number of participants and/or CI crossing the small effect size), and (4) publication bias (i.e., asymmetry in the funnel plot was found). Certainty of the evidence was moderate for eight outcomes, low in four outcomes, and very low for one outcome.

**Table 4:** GRADE (Grading of Recommendations Assessment, Development and Evaluation) assessment for the certainty of evidence

Certainty assessment						No. of participants		Certainty
No. of studies	Risk of bias	Inconsistency	Indirectness	Imprecision	Publication bias	Strength training	Control group	
*V*O_2_max: high load training (follow-up: mean 8.8 weeks)								
11	Not serious	Not serious	Not serious	Serious^a^	Undetected	102	91	⨁⨁⨁◯ Moderate
*V*O_2_max: plyometric training (follow-up: mean 7.7 weeks)								
12	Not serious	Not serious	Not serious	Serious^a^	Undetected	148	130	⨁⨁⨁◯ Moderate
*V*O_2_max: combined training (follow-up: mean 13.1 weeks)								
10	Serious^b^	Not serious	Not serious	Serious^a^	Undetected	107	95	⨁⨁◯◯ Low
v*V*O_2_max: high load training (follow-up: mean 9.2 weeks)								
6	Not serious	Not serious	Not serious	Serious^a^	Undetected	55	44	⨁⨁⨁◯ Moderate
v*V*O_2_max: plyometric training (follow-up: mean 7.2 weeks)								
5	Not serious	Not serious	Not serious	Serious^a^	Undetected	54	45	⨁⨁⨁◯ Moderate
v*V*O_2_max: combined training (follow-up: mean 15.7 weeks)								
6	Serious^b^	Not serious	Not serious	Serious^a^	Undetected	67	58	⨁⨁◯◯ Low
Maximum metabolic steady state: high load training (follow-up: mean 8.5 weeks)								
9	Not serious	Not serious	Not serious	Serious^a^	Undetected	82	72	⨁⨁⨁◯ Moderate
Maximum metabolic steady state: plyometric training (follow-up: mean 7.1 weeks)								
8	Not serious	Not serious	Not serious	Serious^a^	Undetected	110	100	⨁⨁⨁◯ Moderate
Maximum metabolic steady state: combined training (follow-up: mean 18.2 weeks)								
5	Serious^b^	Not serious	Not serious	Serious^a^	Undetected	53	46	⨁⨁◯◯ Low
Sprint capacity: combined training (follow-up: mean 12 weeks)								
5	Serious^b^	Not serious	Not serious	Serious^a^	Undetected	48	45	⨁⨁◯◯ Low
Running performance: high load training (follow-up: mean 8.1 weeks)								
8	Not serious	Not serious	Not serious	Serious^a^	Undetected	72	65	⨁⨁⨁◯ Moderate
Running performance: plyometric training (follow-up: mean 7.4 weeks)								
12	Not serious	Not serious	Not serious	Serious^a^	Undetected	189	169	⨁⨁⨁◯ Moderate
Running performance: combined training (follow-up: mean 14.7 weeks)								
6	Serious^b^	Serious^c^	Not serious	Serious^d^	Detected^c^	59	52	Very low ⨁◯◯◯

*VO*_*2*_*max* maximal oxygen uptake, *vVO*_*2*_*max* velocity at maximal oxygen uptake

^a^Downgraded by one level because *n* < 800 and/or the 95% confidence interval crossed the small effect size

^b^Downgraded by one level because the median PEDro scale is < 6

^c^Downgraded by one level because significant heterogeneity was found

^d^Downgraded by one level because asymmetry in the funnel plot was observed

### ***V***O_2_max

**Fig. 2:**
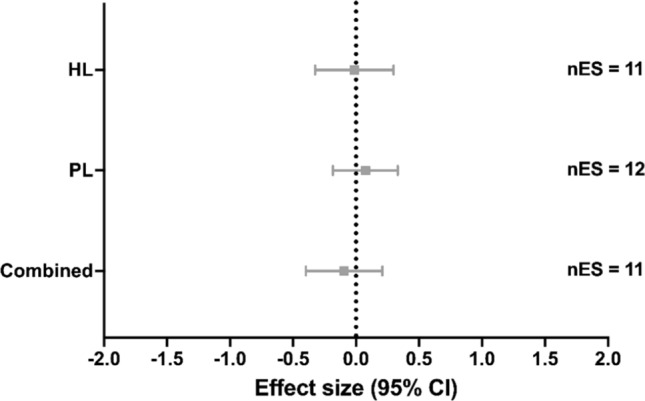
Effect of strength training methods on maximal oxygen uptake. A positive effect size represents beneficial effects after strength training compared with control conditions. *CI* confidence interval, *Combined* high load training, plyometric training and/or submaximal load training, *HL* high load training, *nES* number of effect sizes, *PL* plyometric training

From the studies that measured *V*O_2_max, 11 studies implemented HL [21, 22, 24, 87, 89, 94, 98, 101, 105–107], two studies SubL [85, 97] (not included in the meta-analysis), 12 studies (involving 14 groups) implemented PL [20, 23, 83, 85, 90–92, 95, 96, 99, 100, 104], and ten studies (involving 11 groups) implemented Combined [3, 21, 84, 86, 88, 93, 97, 102, 103, 107]. Compared with control conditions, no significant effects on *V*O_2_max were found with HL training (ES [95% CI] =  − 0.014 [− 0.324 to 0.297], *p* = 0.924, *I*^2^ < 0.001%; Fig. 2), PL (ES [95% CI] = 0.075 [− 0.183 to 0.332], *p* = 0.541, *I*^2^ < 0.001%; Fig. 2) or Combined (ES [95% CI] =  − 0.095 [− 0.398 to 0.208], *p* = 0.499, *I*^2^ < 0.001%; Fig. 2). Meta-regressions and subgroup analyses showed no significant moderating variables for any ST method (all *p* > 0.166; Tables S3–5 of the ESM).

### v***V***O_2_max

**Fig. 3:**
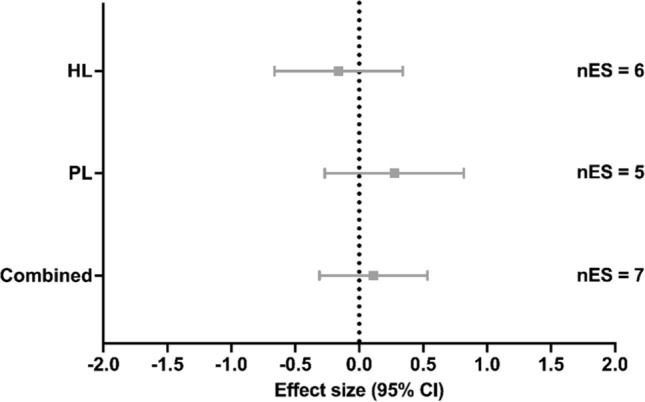
Effect of strength training methods on velocity at maximal oxygen uptake. A positive effect size represents beneficial effects after strength training compared with control conditions. *CI* confidence interval, *Combined* high load training, plyometric training and/or submaximal load training, *HL* high load training, *nES* number of effect sizes, *PL* plyometric training

Among the studies that measured v*V*O_2_max, six studies applied HL [22, 87, 94, 105–107], two studies applied SubL [85, 97] (not included in the meta-analysis), five studies applied PL [23, 83, 85, 91, 108], and six studies (involving seven groups) applied Combined [84, 86, 93, 97, 102, 107]. Compared with the control group, no significant effects on v*V*O_2_max were found with HL training (ES [95% CI] =  − 0.161 [− 0.662 to 0.341], *p* = 0.448, *I*^2^ < 0.001%; Fig. 3), PL (ES [95% CI] = 0.275 [− 0.269 to 0.818], *p* = 0.233, *I*^2^ < 0.001%; Fig. 3) or Combined (ES [95% CI] = 0.112 [− 0.311 to 0.534], *p* = 0.542, *I*^2^ < 0.001%; Fig. 3). The reduced number of studies (i.e., < 8) per each ST method precluded meta-regression and subgroup analyses.

### Maximum Metabolic Steady State

**Fig. 4:**
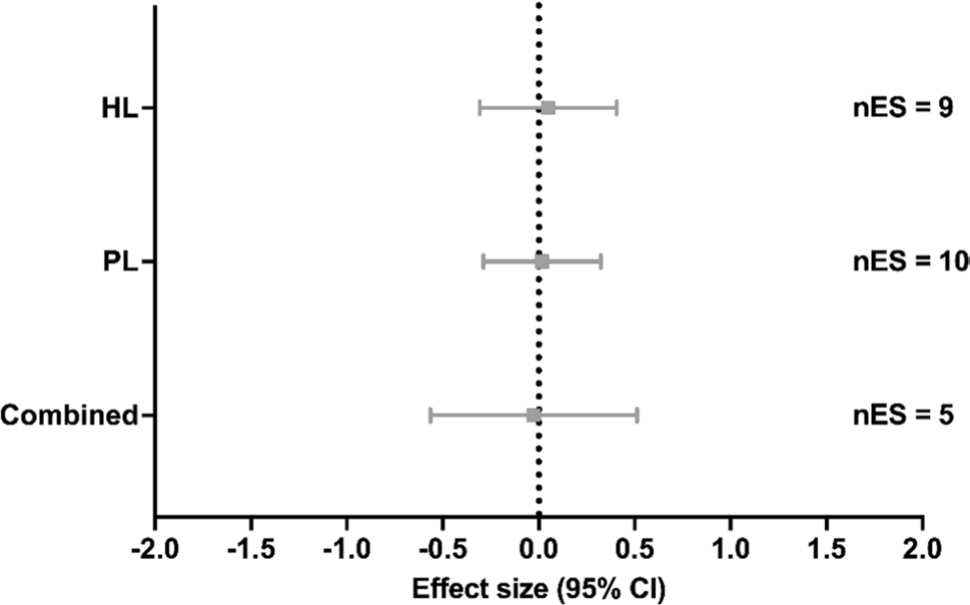
Effect of strength training methods on maximum metabolic steady state. A positive effect size represents beneficial effects after strength training compared with control conditions. *CI* confidence interval, *Combined* high load training, plyometric training, and/or submaximal load training, *HL* high load training, *nES* number of effect sizes, *PL* plyometric training

From the studies that measured MMSS, nine studies implemented HL [22, 24, 87, 94, 101, 105–107, 109], ten groups from eight studies implemented PL [20, 23, 83, 90, 95, 99, 100, 108], and five studies implemented Combined [3, 84, 86, 107, 110]. Compared with the control condition, no significant effects on MMSS were found with HL training (ES [95% CI] = 0.049 [− 0.308 to 0.407], *p* = 0.760, *I*^2^ < 0.001%; Fig. 4), PL (ES [95% CI] = 0.017 [− 0.289 to 0.323], *p* = 0.902, *I*^2^ < 0.001%; Fig. 4) or Combined (ES [95% CI] =  − 0.026 [− 0.564 to 0.513], *p* = 0.902, *I*^2^ < 0.001%; Fig. 4). Meta-regressions and subgroup analyses showed no significant effects of possible moderators in the HL and PL methods (all *p* > 0.181; Tables S6 and S7 of the ESM).

### Sprint Capacity

**Fig. 5:**
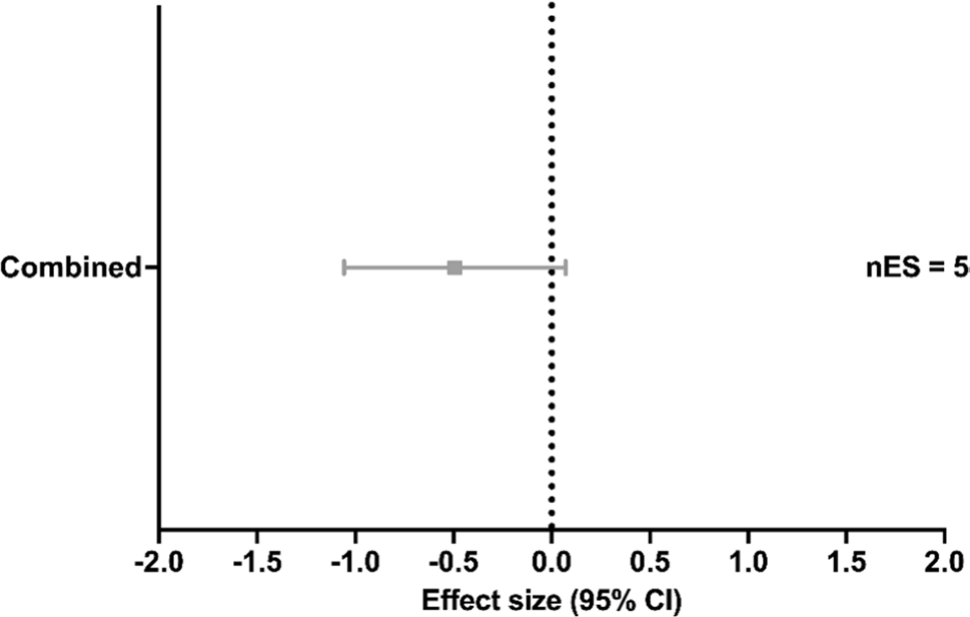
Effect of strength training methods on sprint capacity. A negative effect size represents beneficial effects after strength training compared with control conditions. *CI* confidence interval, *Combined* high load training, plyometric training and/or submaximal load training, *nES* number of effect sizes

Of the studies that measured sprint capacity, two studies applied HL [21, 106], two studies applied PL [92, 112], and five studies applied Combined [3, 21, 86, 93, 111]. Compared with the control condition, no significant effect on sprint capacity was found with Combined training (ES [95% CI] =  − 0.493 [− 1.057 to 0.070], *p* = 0.072, *I*^2^ < 0.001%; Fig. 5).

### Running Performance

**Fig. 6:**
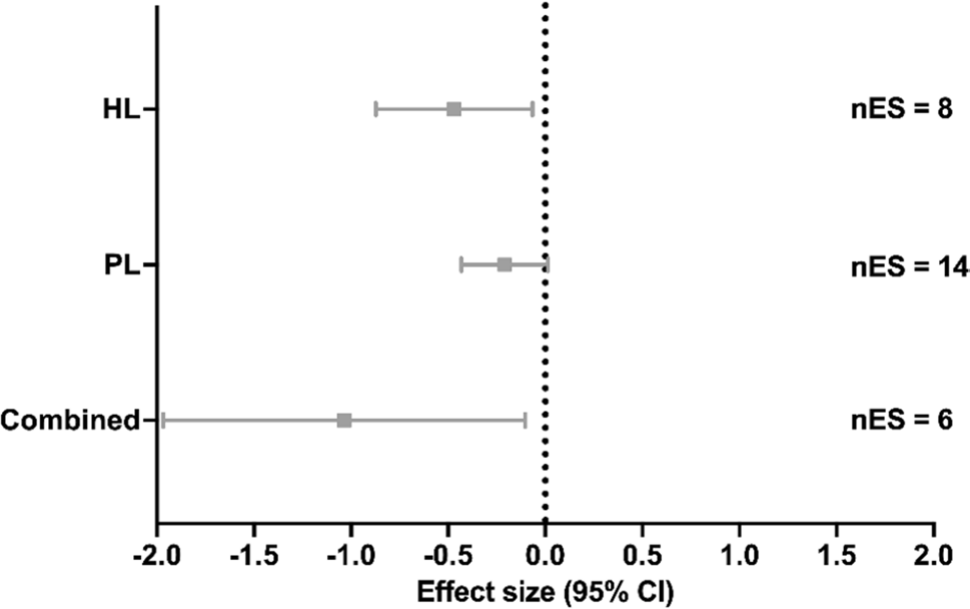
Effect of strength training methods on running performance. A negative effect size represents beneficial effects after strength training compared with control conditions. *CI* confidence interval, *Combined* high load training, plyometric training, and/or submaximal load training, *HL* high load training, *nES* number of effect sizes, *PL* plyometric training

From the studies that measured running performance, eight studies implemented HL [21, 22, 89, 98, 101, 105, 106, 109], two studies implemented SubL [85, 97], 14 groups from 12 studies implemented PL [20, 23, 85, 90–92, 95, 99, 108, 112–114], and six studies implemented Combined [3, 21, 97, 103, 110, 111]. Compared with the control group, a significant moderate effect were found with HL training (ES [95% CI] =  − 0.469 [− 0.872 to − 0.066], *p* = 0.029, *I*^2^ < 0.001%; Fig. 6) and a significant large effect with Combined training but with a significant and moderate level of heterogeneity (ES [95% CI] =  − 1.035 [− 1.967 to − 0.103], *p* = 0.036; *Q*(5) = 15.373, *p* = 0.009, *I*^2^ = 67.475%; Fig. 6). No significant effect on running performance was found with PL training (ES [95% CI] =  − 0.210 [− 0.433 to 0.014], *p* = 0.064, *I*^2^ < 0.001%; Fig. 6). Meta-regressions and subgroup analyses showed no significant effects of possible moderators (all *p* > 0.211, Tables S8 and S9 of the ESM).

## Discussion

The aim of this systematic review with meta-analysis was to analyze the effect of different ST methods (i.e., HL, SubL, PL, and Combined) on performance and its determinants (i.e., *V*O_2_max, v*V*O_2_max, MMSS, and sprint capacity) in middle-distance and long-distance runners. The analyses revealed that, compared with endurance training alone or with ST with very low loads, the HL produced a significant moderate effect on running performance but not PL. Furthermore, when more than two ST methods (i.e., HL, PL and/or SubL) are combined, a significant large effect on running performance is produced. In contrast, no effects on *V*O_2_max, v*V*O_2_max, MMSS, and sprint capacity were found for all ST methods analyzed. These results suggest that HL is an effective method for improving running performance without interfering with other physiological parameters (i.e., *V*O_2_max, v*V*O_2_max, and MMSS), and this effect may be enhanced when PL and HL and/or SubL are combined.

### ***V***O_2_max and v***V***O_2_max

Maximal oxygen uptake is defined as the highest rate at which oxygen can be taken up and utilized by the body during severe exercise [5] and is an important prerequisite for performance in middle-distance and long-distance running [115]. There was no significant effect of any ST method on *V*O_2_max (all *p* > 0.544), which is consistent with previous meta-analyses in endurance athletes [10, 16]. An improvement in *V*O_2_max depends mainly on “upstream factors”, which include all physiological pathways that transfer oxygen from the environment to the blood, pumping it to the periphery and distributing it to and within muscle cells [116, 117]. The short duration of most ST efforts (i.e., exercise duration) probably did not induce an adequate stimulus to these factors. For example, traditional ST with variable resistance elevates oxygen uptake to approximately 45% of *V*O_2_max [118], which is not a sufficient aerobic stimulus to improve *V*O_2_max [119].

Although traditional ST methods may not stimulate *V*O_2_max, they can induce changes in neuromuscular function, musculotendinous stiffness, and muscle fiber type [120], factors that may aid endurance velocity and, by extension, v*V*O_2_max [3, 18]. The measure of v*V*O_2_max is the interaction between *V*O_2_max and running economy [2, 6], influenced by anaerobic and neuromuscular characteristics [121], and can explain differences in performance that *V*O_2_max and running economy alone cannot [6]. Indeed, v*V*O_2_max has been shown to be a good predictor of performance in middle-distance [122, 123] and long-distance running [124–126]. However, we did not find a significant effect of any ST method on v*V*O_2_max (all *p* > 0.479). The lack of an effect of ST methods on v*V*O_2_max may be related to the test protocol used to measure this outcome, particularly the duration of the stages [127]. Protocols with short duration stages and 1.00-km/h incremental changes every minute have been suggested for athletes to reach v*V*O_2_max, resulting in lower work and energy cost than longer duration stages [128]. From a total of 15 studies included in the meta-analysis for v*V*O_2_max, seven studies [22, 23, 84, 91, 97, 105, 106] used protocols with 1.00-km/h increments every minute, or in shorter stages (i.e., 30 s), and four of these studies showed significant effects after PL [91], HL [106], SubL [97], and Combined methods [84, 97]. From the seven studies that applied longer duration stages (i.e., 3 min or more), three found an effect on v*V*O_2_max after PL [83, 108] and HL [87], whereas others found no effect after Combined [86, 93, 102, 107] and HL [107]. These results are in line with the suggestion of a recent meta-analysis to use ramped protocols to elucidate the effects of plyometric jump training on v*V*O_2_max [16]. Considering the above, future research is needed to determine which protocol is valid for detecting v*V*O_2_max adaptations following different ST methods.

### Maximum Metabolic Steady State

Maximum metabolic steady state dictates the boundary between heavy-intensity exercise and severe-intensity exercise [129, 130]. Below MMSS, exercise intensity can reach a steady state of muscle metabolism, whereas above MMSS this state is altered [131], which means increasing the MMSS through training would enable an athlete to achieve a steady state at higher running speeds. Our meta-analysis found no significant effect of any ST method on MMSS (all *p* > 0.760). The findings suggest that the analyzed ST methods do not generate sufficient metabolic impact to improve the MMSS, which typically benefits from training at intensities around this threshold [132]. Even an alternative approach [133] has been explored involving ST with low loads and high repetitions, aiming at a near-threshold intensity, showing different physiological and mechanical responses compared with aerobic training at these intensities. The absence of an effect of ST on the MMSS (and *V*O_2_max) implies that ST may not induce a sufficient stimulus to induce changes in metabolic factors, at least with traditional ST approaches.

### Sprint Capacity

In our meta-analysis, we found no significant effect of Combined on sprint capacity (*p* = 0.072), but not enough studies were found to be able to perform a meta-analysis (i.e., at least three studies) for the other ST methods. Sprint capacity is an important variable because it allows runners to hold a favorable position at the start of a race and to sprint maximally towards its finish [4]. This may be especially relevant in middle-distance events in which the initial and final parts of the race have a higher proportion of sprinting than longer distances. Indeed, a relationship has been found between time achieved in elite male 800-m races with maximum sprint speed in elite male 800-m runners (*R*^2^ = 0.550) [134] and a near-significant relationship in sub-elite female 800-m runners (*R*^2^ = 0.380, *p* = 0.057) [135], but this has not been correlated with changes in 5-km performance [3]. As the capacity to sprint is determined by the application of skeletal muscle force (and not necessarily by anaerobic metabolism) [136], improvements may be because of improved neuromuscular capabilities [3]. However, it is important to mention that all [3, 86, 93, 111] but one [21] of the studies included sprint training in combination with ST, so these possible improvements may be due more to sprint training than to ST. Therefore, research is needed to examine the effect of ST on sprint capacity and its effect on middle-distance and long-distance races.

### Running Performance

#### High Load Training

Interventions with HL improved running performance, including time trial and time to exhaustion measures (ES =  − 0.469 [moderate], *p* = 0.029). In contrast, our meta-analyses indicated no effect of HL on *V*O_2_max, MMSS, and sprint capacity. Given that no improvement in *V*O_2_max or MMSS was found with HL training, following a model that explains performance through metabolic (*V*O_2_max, MMSS, and running economy) and non-metabolic (running economy and sprint capacity) factors [137, 138], it is reasonable to argue that HL could improve performance through non-metabolic factors such as running economy [137]. Indeed, HL can induce non-metabolic (e.g., neuromuscular) adaptations [139] leading toward an improved rate of force development [101, 139]. A larger rate of force development may allow high force levels to be generated at shorter contact times (i.e., at a faster running pace) [140, 141], allow a faster transition from the braking phase to the propulsive phase [140], and allows for quasi-isometric muscle conditions that favor muscle energy costs [141, 142]. Indeed, the rate of force development has been correlated with running economy [101, 140, 142]. Additionally, HL can generate changes in lower limb stiffness [142–144], improving energy storage and release from the lower limbs during running, and this could lead to a reduction in the energy expenditure during running [145] and thus running economy [142–144]. Additionally, increased absolute strength may reduce relative effort at submaximal running speeds, activating a lower number of higher threshold motor units, resulting in a lower energy cost during running, and thus improved running economy [141]. Indeed, a secondary analysis of studies included in this systematic review revealed improved running economy after HL (ES =  − 0.266, *p* = 0.039).

Furthermore, we included time to exhaustion as an indicator of running performance. Two studies [101, 106] measured time to exhaustion at v*V*O_2_max, showing an improvement after HL intervention. Potentially, these improvements are due to an improvement in running economy [94, 101, 106] and anaerobic capacity [106]. Given that the time to exhaustion at severe intensity (i.e., intensity between MMSS and *V*O_2_max) is constrained by a decline in force production [137] and reduced fiber recruitment [146], it is plausible that enhanced rate force development and maximal strength (i.e., 1 RM) could offset the effects of fatigue through enhanced activation of motor neurons and recruitment of muscle fibers [101]. Indeed, while running at v*V*O_2_max, athletes with reduced decline in force production may reduce the increase in energy cost (i.e., greater muscular strength endurance) [147]. Consequently, HL could contribute to the delay of muscle fatigue at this specific intensity [101]. Overall, considering the myriad of factors associated with running performance [137, 138, 148], future studies should elucidate the underlying mechanisms (particularly non-metabolic factors) of the improvement in running performance (and fatigue resistance) following HL interventions.

#### Plyometric Training

Plyometric training may induce neuromuscular adaptations, such as increased motor unit recruitment and improved intermuscular coordination [149]. These neuromuscular improvements have been shown to correlate with improved running economy and anaerobic capacity [137]. Additionally, PL can improve stiffness and compliance (e.g., muscle, tendon, joint) [99, 150]. This mechanism enables greater storage and release of elastic energy within the tendon [150], resulting in reduced muscle energy expenditure [141], and thus improved running economy. Indeed, recent meta-analyses [13, 16] found a significant improvement of running performance after PL. In contrast, our meta-analysis denotes no improvement of running performance after PL (ES =  − 0.210, *p* = 0.064). One possible reason for the discrepancy is that we included a larger (more representative) number of studies in our analysis (*n* = 12) when compared with recent meta-analyses (*n* = 7–10) [13, 16]. However, most of the analyzed studies in previous meta-analyses (e.g., seven of ten) [16] were also included in this analysis. Further, our meta-analysis yielded a nearly significant effect for PL on running performance, with a higher ES compared with a similar meta-analysis that found a favorable running performance effect after PL (ES =  − 0.210 vs − 0.170, respectively) [13]. The reason for the discrepancies between published meta-analyses and our meta-analysis is currently unclear. Possible methodological differences (e.g., inclusion–exclusion criteria; statistical [meta-analytical] approach) may have played a role.

#### Combined

Combined involves incorporating more than one ST method into a training program. Our study revealed that Combined produced a significant large effect on running performance (ES =  − 1.035, *p* = 0.036), producing a greater effect than the use of a single ST method alone. Interestingly, the studies that included the Combined method utilized PL in combination with HL and/or SubL, confirming that including PL with resistance training has a favorable effect on running performance [16] and a greater effect than HL alone. In addition, in a secondary analysis, we found that the Combined method has a greater effect (ES =  − 0.426, *p* = 0.018) on running economy compared with the HL (ES =  − 0.266, *p* = 0.039), SubL (ES =  − 0.365, *p* = 0.131),and PL (ES =  − 0.122, *p* = 0.167) methods used individually. Therefore, we can assume that this improvement in running economy also translates to enhanced running performance. This can be observed in the study by Li et al. [21], which found that HL alone and HL combined with PL both improved running economy and 5-km running performance. However, despite no significant differences between the two groups, the HL with PL group exhibited a higher percentage improvement than HL alone in both running economy (7.68% vs 4.89% at 14.00 km/h) and running performance (2.80% vs 2.09%) [21].

One reason for an increased effect may be that the incorporation of different ST methods can generate a variety of overloads that challenge the neuromuscular system [19] and potentially enhance running performance by eliciting diverse neuromuscular mechanisms. Additionally, the sequence of exercises corresponding to different ST methods within the same training session or in separate sessions may serve different purposes for the force–velocity profile [151]. For example, contrast training (i.e., high load exercises followed by alternating plyometric exercises) could induce post-activation potentiation by improving the speed of plyometric exercises through enhancing both the force and velocity components, whereas traditional training (i.e., low load exercises followed by high load exercises) may primarily develop the force component and not be potentiated by exercises with low loads [151]. However, improvements have been observed in studies where both ST methods were included in the same session [21, 97, 103], as well as in separate sessions [3]. Of note, from the five studies that included SubL, four [3, 103, 110, 111] instructed athletes to perform exercises with maximal velocity intention, and one [97] described the intervention as explosive training. Maximal movement velocity intention at a given load can positively influence neuromuscular adaptations [152], and therefore running performance adaptations.

### Limitations and Strengths

Some limitations of this meta-analysis should be mentioned. First, because of the different composition of each of the ST methods, we decided to perform a separate analysis of each ST method on each of the performance parameters (i.e., *V*O_2_max, v*V*O_2_max, MMSS, sprint capacity, and running performance), which resulted in the SubL group not reaching the minimum number of studies (i.e., three studies) for the main analysis in any of the performance parameters, while HL and PL did not reach the minimum number of studies for sprint capacity. In addition, in some cases, the minimum number of studies (i.e., eight studies) for a moderator analysis was not achieved. Second, high heterogeneity was found for Combined in the analysis for running performance (*p* = 0.009, *I*^2^ = 67.475), probably because different types of ST methods with varying effects were included in this group, and thus their effect on running performance should be interpreted with caution. Finally, in this study, we have focused mainly on aerobic parameters, but the anaerobic component is also a determinant of running performance [3], as well as durability [153]. The strengths of our study are also important to note. To our knowledge, this is the first meta-analysis to analyze the effect of different ST methods on different parameters determining running performance specifically in middle-distance and long-distance runners. Furthermore, we included time to exhaustion as an indicator of running performance allowing us to increase the number of studies in the analysis and to discuss durability.

## Conclusions

In summary, this systematic review with meta-analysis suggests that ST with HL improves running performance measured by a time trial and time to exhaustion. Combining the PL method with HL and/or SubL showed greater improvement in running performance compared with the ST methods alone, while the PL method alone did not enhance running performance. These improvements occurred without changes in *V*O_2_max, v*V*O_2_max, MMSS, and sprint capacity, suggesting that the adaptations are mainly due to non-metabolic factors. These results suggest that middle-distance and long-distance coaches and athletes should consider the inclusion of more than one ST method in their training plan. Future research should aim to analyze and compare the effect of different ST methods combined and separately on running performance, as well as the underlying mechanisms related to these effects.

### Supplementary Information

Below is the link to the electronic supplementary material.

Supplementary file1 (DOCX 2169 kb)

## Declarations

### Availability of data and material

All data generated or analyzed during this study are included in the article as tables, figures, and/or ESM. Any other data requirement can be directed to the corresponding author upon reasonable request.
