# Supplementary material for: The Effect of Strength Training Methods on Middle-Distance and Long-Distance Runners’ Athletic Performance: A Systematic Review with Meta-analysis
Source: Sports Med. 2024 Apr 17;54(7):1801–33. doi: 10.1007/s40279-024-02018-z (PMC11258194; doi:10.1007/s40279-024-02018-z)
Supplement: Supplementary file 1 — Supplementary file1 (DOCX 2169 kb) [file 40279_2024_2018_MOESM1_ESM.docx]

**Electronic Supplementary Material Table S1**

**Article title:** The effect of strength training methods on middle- and long-distance runners’ athletic performance: A systematic review with meta-analysis.

**Author names:** Cristian Llanos-Lagos, Rodrigo Ramirez-Campillo, Jason Moran, Eduardo Sáez de Villarreal

**Affiliation and e-mail of the corresponding author:**

MSc. Cristian Llanos-Lagos

Universidad Pablo de Olavide

Physical Performance Sports Research Center

41704 Sevilla

Spain

Email: cfllalag@alu.upo.es

ORCID: 0009-0003-6906-4977

**Table S1.** Search strategy

| **Database** | **Full search strategy** |
| --- | --- |
| **PubMed** | (("Marathon Running"[Mesh] OR “Running”[Mesh] OR “Distance run*”[Title] OR “run”[Title] OR “Running” [Title] OR “Middle distance*”[Title] OR “Long distance*”[Title] OR “Endurance athlete*” [Title] OR “800 m*” [Title] OR “1500 m*” [Title] OR “3000 m”[Title] OR “5000 m” [Title] OR “5 km” [Title] OR “5km” [Title] OR “10km” [Title] OR “10 km” [Title] OR “Half marathon*”[Title] OR “Cross countr*”[Title] OR “Marathon*”[Title]) AND (“Resistance Training”[Mesh] OR "Plyometric Exercise"[Mesh] OR "Weight Lifting"[Mesh] OR “Strength training”[Title] OR “Weight training” [Title] OR “Resistance training” [Title] OR “Resistance exercise*”[Title] OR “Concurrent training”[Title] OR “Hypertrophy”[Title] OR “Exercise program*”[Title] OR “Weight-bearing” [Title] OR “Muscular strength”[Title] OR “Muscle strength”[Title] OR “Isoinertial”[Title] OR “Plyometric”[Title] OR “Stretch shortening exercise*”[Title] OR “Shortening contraction”[Title] OR “Reactive strength”[Title] OR “Power training”[Title] OR “Weightlifting”[Title] OR “Weight lifting”[Title] OR “Explosive strength”[Title] OR “Ballistic”[Title] OR “Local muscular”[Title] OR “TRX suspension training”[Title] OR “Suspension training”[Title] OR “Free weight training”[Title] OR “Strengthening”[Title] OR “Flywheel”[Title] OR “Eccentric training”[Title] OR “Eccentric contraction”[Title] OR “Eccentric exercis*”[Title] OR “Eccentric-weight”[Title] OR “Eccentric load*”[Title] OR “Negative muscle work”[Title])) AND (("Oxygen Consumption"[Mesh] OR "Adaptation, Physiological"[Mesh] OR “Maximal oxygen*” OR “Maximum oxygen*” OR “Peak oxygen*” OR “VO2max” OR “VO2peak” OR “Aerobic power” OR “Aerobic capacity” OR “Aerobic endurance” OR “Endurance performance” OR “Cardiovascular performance” OR “Cardiorespiratory fitness”) AND/OR (“vVO2max” OR “Velocity at maximal oxygen*” OR “Maximal aerobic velocity” OR “Maximum aerobic velocity” OR “Maximal aerobic speed” OR “Maximum aerobic speed”) AND/OR (“Energy cost” OR “Energetic cost” OR “Caloric cost” OR “Metabolic cost” OR “Mechanical efficiency” OR “Energy cost of running” OR “Running efficiency” OR “Running economy”) AND/OR ("Anaerobic Threshold"[Mesh] OR “Anaerobic threshold” OR “Ventilatory threshold” OR “Gas exchange threshold” OR “Lactate threshold” OR “Lactate” OR “Lactate turnpoint” OR “Lactate minimum speed” OR “Maximal lactate steady state” OR “Onset of blood lactate accumulation” OR “Respiratory compensation point” OR “Critical speed”) AND/OR (“Maximal anaerobic capacity” OR “Anaerobic” OR “Anaerobic power” OR “Anaerobic capacity” OR “Anaerobic speed” OR “Maximal speed”) AND/OR (“Physical endurance”[Mesh] OR “Performance” OR “time trial” OR “Running performance” OR “Distance run*” OR “Time” OR “Running speed” OR “Running time”)) |
| **Web of Sciences (all databases)** | ((TI=(“Distance run*” OR “Run*” OR “Running” OR “Middle distance*” OR “Long distance*” OR “Endurance athlete*” OR “800 m*” OR “1500 m*” OR “3000 m” OR “5000 m” OR “5 km” OR “5km” OR “10km” OR “10 km” OR “Half marathon*” OR “Cross countr*” OR “Marathon*”)) AND TI=(“Strength training” OR “Weight training” OR “Resistance training” OR “Resistance exercise*” OR “Concurrent training” OR “Hypertrophy” OR “Exercise program*” OR “Weight-bearing” OR “Muscular strength” OR “Muscle strength” OR “Isoinertial” OR “Plyometric” OR “Stretch shortening exercise*” OR “Shortening contraction” OR “Reactive strength” OR “Power training” OR “Weightlifting” OR “Weight lifting” OR “Explosive strength” OR “Ballistic” OR “Local muscular” OR “TRX suspension training” OR “Suspension training” OR “Free weight training” OR “Strengthening” OR “Flywheel” OR “Eccentric training” OR “Eccentric contraction” OR “Eccentric exercis*” OR “Eccentric-weight” OR “Eccentric load*” OR “Negative muscle work”)) AND TS=(“Maximal oxygen*” OR “Maximum oxygen*” OR “Peak oxygen*” OR “VO2max” OR “VO2peak” OR “Aerobic power” OR “Aerobic capacity” OR “Aerobic endurance” OR “Endurance performance” OR “Cardiovascular performance” OR “Cardiorespiratory fitness” OR “vVO2max” OR “Velocity at maximal oxygen*” OR “Maximal aerobic velocity” OR “Maximum aerobic velocity” OR “Maximal aerobic speed” OR “Maximum aerobic speed” OR “Energy cost” OR “Energetic cost” OR “Caloric cost” OR “Metabolic cost” OR “Mechanical efficiency” OR “Energy cost of running” OR “Running efficiency” OR “Running economy” OR “Anaerobic threshold” OR “Ventilatory threshold” OR “Gas exchange threshold” OR “Lactate threshold” OR “Lactate” OR “Lactate turnpoint” OR “Lactate minimum speed” OR “Maximal lactate steady state” OR “Onset of blood lactate accumulation” OR “Respiratory compensation point” OR “Critical speed” OR “Maximal anaerobic capacity” OR “Anaerobic” OR “Anaerobic power” OR “Anaerobic capacity” OR “Anaerobic speed” OR “Maximal speed” OR “Performance” OR “Time trial” OR “Running performance” OR “Distance run*” OR “Time” OR “Running speed” OR “Running time”) |
| **Scopus** | TITLE (“Distance run*” OR “Run*” OR “Running” OR “Middle distance*” OR “Long distance*” OR “Endurance athlete*” OR “800 m*” OR “1500 m*” OR “3000 m” OR “5000 m” OR “5 km” OR “5km” OR “10km” OR “10 km” OR “Half marathon*” OR “Cross countr*” OR “Marathon*”) AND TITLE (“Strength training” OR “Weight training” OR “Resistance training” OR “Resistance exercise*” OR “Concurrent training” OR “Hypertrophy” OR “Exercise program*” OR “Weight-bearing” OR “Muscular strength” OR “Muscle strength” OR “Isoinertial” OR “Plyometric” OR “Stretch shortening exercise*” OR “Shortening contraction” OR “Reactive strength” OR “Power training” OR “Weightlifting” OR “Weight lifting” OR “Explosive strength” OR “Ballistic” OR “Local muscular” OR “TRX suspension training” OR “Suspension training” OR “Free weight training” OR “Strengthening” OR “Flywheel” OR “Eccentric training” OR “Eccentric contraction” OR “Eccentric exercis*” OR “Eccentric-weight” OR “Eccentric load*” OR “Negative muscle work”) AND ALL (“Maximal oxygen*” OR “Maximum oxygen*” OR “Peak oxygen*” OR “VO2max” OR “VO2peak” OR “Aerobic power” OR “Aerobic capacity” OR “Aerobic endurance” OR “Endurance performance” OR “Cardiovascular performance” OR “Cardiorespiratory fitness” OR “vVO2max” OR “Velocity at maximal oxygen*” OR “Maximal aerobic velocity” OR “Maximum aerobic velocity” OR “Maximal aerobic speed” OR “Maximum aerobic speed” OR “Energy cost” OR “Energetic cost” OR “Caloric cost” OR “Metabolic cost” OR “Mechanical efficiency” OR “Energy cost of running” OR “Running efficiency” OR “Running economy” OR “Anaerobic threshold” OR “Ventilatory threshold” OR “Gas exchange threshold” OR “Lactate threshold” OR “Lactate” OR “Lactate turnpoint” OR “Lactate minimum speed” OR “Maximal lactate steady state” OR “Onset of blood lactate accumulation” OR “Respiratory compensation point” OR “Critical speed” OR “Maximal anaerobic capacity” OR “Anaerobic” OR “Anaerobic power” OR “Anaerobic capacity” OR “Anaerobic speed” OR “Maximal speed” OR “Performance” OR “Time trial” OR “Running performance” OR “Distance run*” OR “Time” OR “Running speed” OR “Running time”) |
| **SportDiscus** | ( TI (“Distance run*” OR “Run*” OR “Running” OR “Middle distance*” OR “Long distance*” OR “Endurance athlete*” OR “800 m*” OR “1500 m*” OR “3000 m” OR “5000 m” OR “5 km” OR “5km” OR “10km” OR “10 km” OR “Half marathon*” OR “Cross countr*” OR “Marathon*”) ) AND ( TI (“Strength training” OR “Weight training” OR “Resistance training” OR “Resistance exercise*” OR “Concurrent training” OR “Hypertrophy” OR “Exercise program*” OR “Weight-bearing” OR “Muscular strength” OR “Muscle strength” OR “Isoinertial” OR “Plyometric” OR “Stretch shortening exercise*” OR “Shortening contraction” OR “Reactive strength” OR “Power training” OR “Weightlifting” OR “Weight lifting” OR “Explosive strength” OR “Ballistic” OR “Local muscular” OR “TRX suspension training” OR “Suspension training” OR “Free weight training” OR “Strengthening” OR “Flywheel” OR “Eccentric training” OR “Eccentric contraction” OR “Eccentric exercis*” OR “Eccentric-weight” OR “Eccentric load*” OR “Negative muscle work”) ) AND TX ( (“Maximal oxygen*” OR “Maximum oxygen*” OR “Peak oxygen*” OR “VO2max” OR “VO2peak” OR “Aerobic power” OR “Aerobic capacity” OR “Aerobic endurance” OR “Endurance performance” OR “Cardiovascular performance” OR “Cardiorespiratory fitness” OR “vVO2max” OR “Velocity at maximal oxygen*” OR “Maximal aerobic velocity” OR “Maximum aerobic velocity” OR “Maximal aerobic speed” OR “Maximum aerobic speed” OR “Energy cost” OR “Energetic cost” OR “Caloric cost” OR “Metabolic cost” OR “Mechanical efficiency” OR “Energy cost of running” OR “Running efficiency” OR “Running economy” OR “Anaerobic threshold” OR “Ventilatory threshold” OR “Gas exchange threshold” OR “Lactate threshold” OR “Lactate” OR “Lactate turnpoint” OR “Lactate minimum speed” OR “Maximal lactate steady state” OR “Onset of blood lactate accumulation” OR “Respiratory compensation point” OR “Critical speed” OR “Maximal anaerobic capacity” OR “Anaerobic” OR “Anaerobic power” OR “Anaerobic capacity” OR “Anaerobic speed” OR “Maximal speed” OR “Performance” OR “Time trial” OR “Running performance” OR “Distance run*” OR “Time” OR “Running speed” OR “Running time”) ) |

Mesh: Medical Subject Headings; TI: Title; TS: Topic.

**Electronic Supplementary Material Table S2**

**Article title:** The effect of strength training methods on middle- and long-distance runners’ athletic performance: A systematic review with meta-analysis.

**Author names:** Cristian Llanos-Lagos, Rodrigo Ramirez-Campillo, Jason Moran, Eduardo Sáez de Villarreal

**Affiliation and e-mail of the corresponding author:**

MSc. Cristian Llanos-Lagos

Universidad Pablo de Olavide

Physical Performance Sports Research Center

41704 Sevilla

Spain

Email: cfllalag@alu.upo.es

ORCID: 0009-0003-6906-4977

**Table S2.** Physiotherapy Evidence Database (PEDro) scale.

| **Authors, year** | **N1** | **N2** | **N3** | **N4** | **N5** | **N6** | **N7** | **N8** | **Total** | **Risk of bias** |
| --- | --- | --- | --- | --- | --- | --- | --- | --- | --- | --- |
| Ache-Dias *et al.* [83] | Yes | 1 |  | 1 | 1 | 1 | 1 | 1 | 6 | Low risk |
| Bachero-Mena *et al.* [111] | Yes |  |  | 1 |  | 1 | 1 | 1 | 4 | Moderate risk |
| Beattie *et al.* [84] | Yes |  |  | 1 |  | 1 | 1 | 1 | 4 | Moderate risk |
| Berryman *et al.,* 2010 [85] | Yes | 1 |  | 1 | 1 | 1 | 1 | 1 | 6 | Low risk |
| Bertuzzi *et al.* [22] | Yes | 1 |  | 1 | 1 | 1 | 1 | 1 | 6 | Low risk |
| Blagrove *et al.* [86] | Yes | 1 |  | 1 |  | 1 | 1 | 1 | 5 | Moderate risk |
| Damasceno *et al.* [87] | Yes | 1 | 1 | 1 | 1 | 1 | 1 | 1 | 7 | Low risk |
| do Carmo *et al.* [23] | Yes | 1 |  | 1 | 1 | 1 | 1 | 1 | 6 | Low risk |
| Ferrauti *et al.* [24] | Yes | 1 |  | 1 | 1 | 1 | 1 | 1 | 6 | Low risk |
| Filipas *et al.* [20] | Yes | 1 | 1 | 1 | 1 | 1 | 1 | 1 | 7 | Low risk |
| García-Pinillos *et al.* [113] | Yes |  |  | 1 | 1 | 1 | 1 | 1 | 5 | Moderate risk |
| Hamilton *et al.* [108] | Yes | 1 |  | 1 | 1 | 1 | 1 | 1 | 6 | Low risk |
| Johnston *et al.* [88] | Yes | 1 |  | 1 | 1 | 1 | 1 | 1 | 6 | Low risk |
| Karsten *et al.* [109] | Yes | 1 |  | 1 | 1 | 1 | 1 | 1 | 6 | Low risk |
| Kelly *et al.* [89] | Yes | 1 |  | 1 | 1 | 1 | 1 | 1 | 6 | Low risk |
| Li *et al.* [21] | Yes |  |  | 1 | 1 | 1 | 1 | 1 | 5 | Moderate risk |
| Lum *et al.* [90] | Yes | 1 |  | 1 | 1 | 1 | 1 | 1 | 6 | Low risk |
| Lum *et al.* [91] | Yes | 1 |  | 1 | 1 | 1 | 1 | 1 | 6 | Low risk |
| Lundstrom *et al.* [92] | Yes | 1 |  | 1 | 1 | 1 | 1 | 1 | 6 | Low risk |
| Machado *et al.* [114] | Yes | 1 |  | 1 | 1 | 1 | 1 | 1 | 6 | Low risk |
| Mikkola *et al.* [93] | Yes |  |  | 1 | 1 | 1 | 1 | 1 | 5 | Moderate risk |
| Mikkola *et al.* [107] | Yes |  |  | 1 | 1 | 1 | 1 | 1 | 5 | Moderate risk |
| Millet *et al.* [94] | Yes | 1 |  | 1 | 1 | 1 | 1 | 1 | 6 | Low risk |
| Paavolainen *et al* [3] | Yes |  |  | 1 |  | 1 | 1 | 1 | 4 | Moderate risk |
| Piacentini *et al.* [74] | Yes | 1 |  | 1 | 1 | 1 | 1 | 1 | 6 | Low risk |
| Ramírez-Campillo *et al.* [112] | Yes | 1 | 1 | 1 | 1 | 1 | 1 | 1 | 7 | Low risk |
| Saunders *et al.* [96] | Yes | 1 |  | 1 | 1 | 1 | 1 | 1 | 6 | Low risk |
| Schumann *et al.* [110] | Yes |  |  | 1 | 1 | 1 | 1 | 1 | 5 | Moderate risk |
| Sedano *et al.* [97] | Yes | 1 |  | 1 | 1 | 1 | 1 | 1 | 6 | Low risk |
| Skovgaard *et al.* [98] | Yes | 1 |  | 1 | 1 | 1 | 1 | 1 | 6 | Low risk |
| Spurrs *et al.* [99] | Yes | 1 |  | 1 | 1 | 1 | 1 | 1 | 6 | Low risk |
| Štohanzl *et al.* [100] | Yes | 1 |  | 1 | 1 | 1 | 1 | 1 | 6 | Low risk |
| Støren *et al.* [101] | Yes | 1 |  | 1 | 1 | 1 | 1 | 1 | 6 | Low risk |
| Taipale *et al.* [102] | Yes |  |  | 1 | 1 | 1 | 1 | 1 | 5 | Moderate risk |
| Trowell *et al.* [103] | Yes | 1 | 1 | 1 | 1 | 1 | 1 | 1 | 7 | Low risk |
| Turner *et al.* [104] | Yes | 1 |  | 1 | 1 | 1 | 1 | 1 | 6 | Low risk |
| Vikmoen *et al.* [105] | Yes | 1 |  | 1 | 1 | 1 | 1 | 1 | 6 | Low risk |

Vorup *et al.* [106]sures were available received the treatment or control condition as allocated or, where this was not the case, data for at least one key outcome was analysed by “intention to treat”; N7: the results of between-group statistical comparisons are reported for at least one key outcome; N8: the study provides both point measures and measures of variability for at least one key outcome; Risk of bias: ≥ 6 points = “low risk”, 4 to 5 points = “moderate risk”, and ≤ 3 points = “high risk”. Items 5 to 7 of the original scale were removed due to the infrequency of blinding of subjects, evaluators, and researchers in supervised exercise interventions.

**Electronic Supplementary Material Table S3**

**Article title:** The effect of strength training methods on middle- and long-distance runners’ athletic performance: A systematic review with meta-analysis.

**Author names:** Cristian Llanos-Lagos, Rodrigo Ramirez-Campillo, Jason Moran, Eduardo Sáez de Villarreal

**Affiliation and e-mail of the corresponding author:**

MSc. Cristian Llanos-Lagos

Universidad Pablo de Olavide

Physical Performance Sports Research Center

41704 Sevilla

Spain

Email: cfllalag@alu.upo.es

ORCID: 0009-0003-6906-4977

**Table S3.** Results of meta-regression and subgroup analyses in search of possible moderators of high load training on VO_2_max.

| **High load training** | *n* groups | *β_0_* Hedges' *g* (SE) | CI 95% | *t_0_*(*df*)*, p* value | *β_1_* Hedges' *g* (SE) | CI 95% | *t_1_*(*df*)*, p* value | *F*(*df_1_, df_2_*)*, p* value |
| --- | --- | --- | --- | --- | --- | --- | --- | --- |
| Subject characteristics |  |  |  |  |  |  |  |  |
| Sex | 11 |  |  |  |  |  |  | *F*(2,8) = 2.268, *p* = 0.166 |
| Male | 2 | -0.241 (0.177) | -0.649 to 0.167 | *t*(8) = -1.361, *p* = 0.211 |  |  |  |  |
| Female | 7 | 0.466 (0.330) | -0.294 to 1.226 | *t*(8) = -1.415, *p* = 0.195 | 0.707 (0.374) | -0.156 to 1.570 | *t*(8) = 1.890, *p* = 0.095 |  |
| Male-Female | 2 | 0.257 (0.309) | -0.455 to 0.969 | *t*(8) = 0.832, *p* = 0.430 | 0.498 (0.356) | -0.323 to 1.319 | *t*(8) = 1.398, *p* = 0.200 |  |
| Age | 11 | -0.602 (0.697) | -2.178 to 0.973 | *t*(9) = -0.865, *p* = 0.410 | 0.019 (0.022) | -0.031 to 0.069 | *t*(9) = 0.863, *p* = 0.411 | *F*(1,9) = 0.744, *p* = 0.411 |
| Body mass | 11 | 0.559 (1.561) | -2.973 to 4.091 | *t*(9) = 0.358, *p* = 0.728 | -0.008 (0.022) | -0.059 to 0.042 | *t*(9) = -0.368, *p* = 0.721 | *F*(1,9) = 0.136, *p* = 0.721 |
| Height | 10 | 7.323 (5.075) | -4.379 to 19.025 | *t*(8) = 1.443, *p* = 0.187 | -0.042 (0.029) | -0.109 to 0.025 | *t*(8) = -1.458, *p* = 0.183 | *F*(1,8) = 2.126, *p* = 0.183 |
| Initial VO_2_max | 11 | 1.536 (1.073) | -0.890 to 3.963 | *t*(9) = 1.432, *p* = 0.186 | -0.028 (0.019) | -0.070 to 0.015 | *t*(9) = -1.457, *p* = 0.179 | *F*(1,9) = 2.124, *p* = 0.179 |
| Performance level | 11 |  |  |  |  |  |  | *F*(2,8) = 1.080, *p* = 0.384 |
| Moderately trained | 5 | -0.099 (0.273) | -0.728 to 0.531 | *t*(8) = -0.362, *p* = 0.727 |  |  |  |  |
| Well trained | 4 | 0.188 (0.202) | -0.277 to 0.653 | *t*(8) = 0.931, *p* = 0.379 | 0.287 (0.339) | -0.496 to 1.069 | *t*(8) = 0.845, *p* = 0.423 |  |
| Highly trained | 2 | -0.293 (0.271) | -0.918 to 0.332 | *t*(8) = -1.082, *p* = 0.311 | -0.195 (0.385) | -1.081 to 0.692 | *t*(8) = -0.506, *p* = 0.627 |  |
| Strength training experience | 9 |  |  |  |  |  |  | *F*(1,6) = 1.919, *p* = 0.215 |
| No | 7 | 0.188 (0.173) | -0.234 to 0.610 | *t*(6) = 1.091, *p* = 0.317 |  |  |  |  |
| Yes | 2 | -0.532 (0.490) | -1.731 to 0.668 | *t*(6) = -1.085, *p* = 0.320 | -0.720 (0.520) | -1.992 to 0.552 | *t*(6) = -1.385, *p* = 0.215 |  |
| Strength training intervention |  |  |  |  |  |  |  |  |
| Weeks | 11 | -0.103 (0.646) | -1.563 to 1.358 | *t*(9) = -0.159, *p* = 0.877 | 0.010 (0.072) | -0.152 to 0.173 | *t*(9) = 0.141, *p* = 0.891 | *F*(1,9) = 0.020, *p* = 0.891 |
| Sessions per week | 11 | 0.133 (0.525) | -1.055 to 1.322 | *t*(9) = 0.253, *p* = 0.806 | -0.059 (0.204) | -0.521 to 0.402 | *t*(9) = -0.290, *p* = 0.779 | *F*(1,9) = 0.084, *p* = 0.779 |
| Total sessions | 11 | 0.028 (0.492) | -1.084 to 1.140 | *t*(9) = 0.057, *p* = 0.956 | -0.002 (0.022) | -0.051 to 0.047 | *t*(9) = -0.088, *p* = 0.932 | *F*(1,9) = 0.008, *p* = 0.932 |

In the subgroup analysis (categorical variables), the first variable of the category was considered as the reference. n groups: number of experimental groups; SE, standard error; CI, confidence Interval; df, degree of freedom.

**Electronic Supplementary Material Table S4**

**Article title:** The effect of strength training methods on middle- and long-distance runners’ athletic performance: A systematic review with meta-analysis.

**Author names:** Cristian Llanos-Lagos, Rodrigo Ramirez-Campillo, Jason Moran, Eduardo Sáez de Villarreal

**Affiliation and e-mail of the corresponding author:**

MSc. Cristian Llanos-Lagos

Universidad Pablo de Olavide

Physical Performance Sports Research Center

41704 Sevilla

Spain

Email: cfllalag@alu.upo.es

ORCID: 0009-0003-6906-4977

**Table S4.** Results of meta-regression and subgroup analyses in search of possible moderators of plyometric training on VO_2_max.

| **Plyometric training** | *n* groups | *β_0_* Hedges' *g* (SE) | CI 95% | *t_0_*(*df*)*, p* value | *β_1_* Hedges' *g* (SE) | CI 95% | *t_1_*(*df*)*, p* value | *F*(*df_1_, df_2_*)*, p* value |
| --- | --- | --- | --- | --- | --- | --- | --- | --- |
| Subject characteristics |  |  |  |  |  |  |  |  |
| Sex | 14 |  |  |  |  |  |  | *F*(2,11) = 0.527, *p* = 0.605 |
| Male | 7 | -0.011 (0.166) | -0.377 to 0.354 | *t*(11) = -0.068, *p* = 0.947 |  |  |  |  |
| Female | 2 | -0.052 (0.354) | -0.830 to 0.727 | *t*(11) = -0.147, *p* = 0.886 | -0.040 (0.391) | -0.900 to 0.819 | *t*(11) = -0.104, *p* = 0.919 |  |
| Male-Female | 5 | 0.233 (0.196) | -0.197 to 0.663 | *t*(11) = 1.193, *p* = 0.258 | 0.245 (0.256) | -0.320 to 0.809 | *t*(11) = 0.954, *p* = 0.361 |  |
| Age | 14 | 0.049 (0.827) | -1.752 to 1.850 | *t*(12) = 0.060, *p* = 0.953 | 0.001 (0.027) | -0.058 to 0.060 | *t*(12) = 0.031, *p* = 0.976 | *F*(1,12) = 0.001, *p* = 0.976 |
| Body mass | 14 | 1.177 (2.659) | -4.618 to 6.971 | *t*(12) = 0.443, *p* = 0.666 | -0.016 (0.039) | -0.101 to 0.069 | *t*(12) = -0.415, *p* = 0.686 | *F*(1,12) = 0.172, *p* = 0.686 |
| Height | 10 | -0.101 (0.982) | -2.365 to 2.163 | *t*(8) = -0.103, *p* = 0.921 | 0.001 (0.006) | -0.013 to 0.015 | *t*(8) = 0.169, *p* = 0.870 | *F*(1,8) = 0.029, *p* = 0.870 |
| Initial VO_2_max | 14 | 0.270 (0.696) | -1.247 to 1.787 | *t*(12) = 0.388, *p* = 0.705 | -0.004 (0.013) | -0.031 to 0.024 | *t*(12) = -0.285, *p* = 0.781 | *F*(1,12) = 0.081, *p* = 0.781 |
| Performance level | 14 |  |  |  |  |  |  | *F*(2,11) = 0.118, *p* = 0.890 |
| Moderately trained | 5 | 0.061 (0.210) | -0.400 to 0.522 | *t*(11) = 0.292, *p* = 0.776 |  |  |  |  |
| Well trained | 6 | 0.132 (0.179) | -0.262 to 0.526 | *t*(11) = 0.736, *p* = 0.477 | 0.071 (0.276) | -0.536 to 0.677 | *t*(11) = 0.256, *p* = 0.803 |  |
| Highly trained | 3 | -0.014 (0.246) | -0.556 to 0.528 | *t*(11) = -0.057, *p* = 0.956 | -0.075 (0.323) | -0.787 to 0.637 | *t*(11) = -0.232, *p* = 0.820 |  |
| Strength training experience | 10 |  |  |  |  |  |  | *F*(1,8) = 0.011, *p* = 0.918 |
| No | 8 | 0.045 (0.154) | -0.311 to 0.401 | *t*(8) = 0.292, *p* = 0.778 |  |  |  |  |
| Yes | 2 | 0.006 (0.336) | -0.770 to 0.782 | *t*(8) = 0.017, *p* = 0.987 | -0.039 (0.370) | -0.893 to 0.814 | *t*(8) = -0.106, *p* = 0.918 |  |
| Strength training intervention |  |  |  |  |  |  |  |  |
| Weeks | 14 | -0.204 (0.521) | -1.340 to 0.931 | *t*(12) = -0.392, *p* = 0.702 | 0.036 (0.065) | -0.107 to 0.179 | *t*(12) = 0.550, *p* = 0.592 | *F*(1,12) = 0.303, *p* = 0.592 |
| Sessions per week | 14 | 0.243 (0.341) | -0.500 to 0.985 | *t*(12) = 0.713, *p* = 0.490 | -0.093 (0.177) | -0.479 to 0.292 | *t*(12) = -0.526, *p* = 0.608 | *F*(1,12) = 0.277, *p* = 0.608 |
| Total sessions | 14 | 0.213 (0.348) | -0.546 to 0.972 | *t*(12) = 0.611, *p* = 0.552 | -0.010 (0.024) | -0.062 to 0.042 | *t*(12) = -0.422, *p* = 0.681 | *F*(1,12) = 0.178, *p* = 0.681 |

In the subgroup analysis (categorical variables), the first variable of the category was considered as the reference. n groups: number of experimental groups; SE, standard error; CI, confidence Interval; df, degree freedom.

**Electronic Supplementary Material Table S5**

**Article title:** The effect of strength training methods on middle- and long-distance runners’ athletic performance: A systematic review with meta-analysis.

**Author names:** Cristian Llanos-Lagos, Rodrigo Ramirez-Campillo, Jason Moran, Eduardo Sáez de Villarreal

**Affiliation and e-mail of the corresponding author:**

MSc. Cristian Llanos-Lagos

Universidad Pablo de Olavide

Physical Performance Sports Research Center

41704 Sevilla

Spain

Email: cfllalag@alu.upo.es

ORCID: 0009-0003-6906-4977

**Table S5**. Results of meta-regression and subgroup analyses in search of possible moderators of combined training on VO_2_max.

| **Combined training** | *n* groups | *β_0_* Hedges' *g* (SE) | CI 95% | *t_0_*(*df*)*, p* value | *β_1_* Hedges' *g* (SE) | CI 95% | *t_1_*(*df*)*, p* value | *F*(*df_1_, df_2_*)*, p* value |
| --- | --- | --- | --- | --- | --- | --- | --- | --- |
| Subject characteristics |  |  |  |  |  |  |  |  |
| Sex | 11 |  |  |  |  |  |  | *F*(2,8) = 0.392, *p* = 0.688 |
| Male | 5 | -0.209 (0.217) | -0.710 to 0.291 | *t*(8) = -0.964, *p* = 0.363 |  |  |  |  |
| Female | 2 | -0.193 (0.345) | -0.988 to 0.603 | *t*(8) = -0.559, *p* = 0.592 | 0.016 (0.408) | -0.923 to 0.956 | *t*(8) = 0.040, *p* = 0.969 |  |
| Male-Female | 4 | 0.037 (0.203) | -0.430 to 0.504 | *t*(8) = 0.185, *p* = 0.858 | 0.247 (0.297) | -0.438 to 0.931 | *t*(8) = 0.831, *p* = 0.430 |  |
| Age | 11 | 0.043 (0.509) | -1.108 to 1.194 | *t*(9) = 0.085, *p* = 0.934 | -0.005 (0.019) | -0.049 to 0.038 | *t*(9) = -0.283, *p* = 0.784 | *F*(1,9) = 0.080, *p* = 0.784 |
| Body mass | 11 | 0.038 (1.094) | -2.437 to 2.513 | *t*(9) = 0.035, *p* = 0.973 | -0.002 (0.016) | -0.039 to 0.035 | *t*(9) = -0.123, *p* = 0.905 | *F*(1,9) = 0.015, *p* = 0.905 |
| Height | 11 | -0.197 (4.313) | -9.954 to 9.560 | *t*(9) = -0.046, *p* = 0.965 | 0.001 (0.025) | -0.055 to 0.056 | *t*(9) = 0.024, *p* = 0.982 | *F*(1,9) = 0.001, *p* = 0.982 |
| Initial VO_2_max | 11 | -1.235 (1.057) | -3.627 to 1.157 | *t*(9) = -1.168, *p* = 0.273 | 0.019 (0.018) | -0.021 to 0.060 | *t*(9) = 1.087, *p* = 0.305 | *F*(1,9) = 1.181, *p* = 0.305 |
| Performance level | 11 |  |  |  |  |  |  | *F*(2,8) = 0.902, *p* = 0.443 |
| Moderately trained | 3 | -0.364 (0.275) | -0.988 to 0.270 | *t*(8) = -1.324, *p* = 0.222 |  |  |  |  |
| Well trained | 1 | -0.386 (0.539) | -1.629 to 0.856 | *t*(8) = -0.717, *p* = 0.494 | -0.022 (0.605) | -1.417 to 1.373 | *t*(8) = -0.037, *p* = 0.972 |  |
| Highly trained | 7 | 0.027 (0.164) | -0.351 to 0.404 | *t*(8) = 0.163, *p* = 0.874 | 0.391 (0.320) | -0.347 to 1.129 | *t*(8) = 1.222, *p* = 0.257 |  |
| Strength training experience | 6 |  |  |  |  |  |  | *F*(1,4) = 0.395, *p* = 0.564 |
| No | 4 | 0.012 (0.217) | -0.591 to 0.616 | *t*(4) = 0.056, *p* = 0.958 |  |  |  |  |
| Yes | 2 | -0.255 (0.365) | -1.267 to 0.758 | *t*(4) = -0.698, *p* = 0.524 | -0.267 (0.424) | -1.445 to 0.912 | *t*(4) = -0.628, *p* = 0.564 |  |
| Strength training intervention |  |  |  |  |  |  |  |  |
| Weeks | 11 | -0.060 (0.542) | -1.288 to 1.167 | *t*(9) = -0.111, *p* = 0.914 | -0.003 (0.050) | -0.117 to 0.110 | *t*(9) = -0.067, *p* = 0.948 | *F*(1,9) = 0.004, *p* = 0.948 |
| Sessions per week | 11 | 0.016 (0.504) | -1.125 to 1.157 | *t*(9) = 0.032, *p* = 0.975 | -0.051 (0.223) | -0.554 to 0.452 | *t*(9) = -0.229, *p* = 0.824 | *F*(1,9) = 0.053, *p* = 0.824 |
| Total sessions | 11 | 0.270 (0.983) | -1.953 to 2.492 | *t*(9) = 0.274, *p* = 0.790 | -0.016 (0.044) | -0.115 to 0.082 | *t*(9) = -0.375, *p* = 0.716 | *F*(1,9) = 0.141, *p* = 0.716 |

In the subgroup analysis (categorical variables), the first variable of the category was considered as the reference. n groups: number of experimental groups; SE, standard error; CI, confidence Interval; df, degree freedom.

**Electronic Supplementary Material Table S6**

**Article title:** The effect of strength training methods on middle- and long-distance runners’ athletic performance: A systematic review with meta-analysis.

**Author names:** Cristian Llanos-Lagos, Rodrigo Ramirez-Campillo, Jason Moran, Eduardo Sáez de Villarreal

**Affiliation and e-mail of the corresponding author:**

MSc. Cristian Llanos-Lagos

Universidad Pablo de Olavide

Physical Performance Sports Research Center

41704 Sevilla

Spain

Email: cfllalag@alu.upo.es

ORCID: 0009-0003-6906-4977

**Table S6**. Results of meta-regression and subgroup analyses in search of possible moderators of high load training on maximum metabolic steady state.

| **High load training** | *n* groups | *β_0_* Hedges' *g* (SE) | CI 95% | *t_0_*(*df*)*, p* value | *β_1_* Hedges' *g* (SE) | CI 95% | *t_1_*(*df*)*, p* value | *F*(*df_1_, df_2_*)*, p* value |
| --- | --- | --- | --- | --- | --- | --- | --- | --- |
| Subject characteristics |  |  |  |  |  |  |  |  |
| Sex | 9 |  |  |  |  |  |  | *F*(2,6) = 0.222, *p* = 0.807 |
| Male | 5 | 0.134 (0.215) | -0.393 to 0.661 | *t*(6) = 0.624, *p* = 0.556 |  |  |  |  |
| Female | 1 | -0.175 (0.445) | -1.263 to 0.913 | *t*(6) = -0.393, *p* = 0.708 | -0.309 (0.494) | -1.518 to 0.900 | *t*(6) = -0.626, *p* = 0.555 |  |
| Male-Female | 3 | 0.002 (0.258) | -0.630 to 0.634 | *t*(6) = 0.008, *p* = 0.994 | -0.132 (0.336) | -0.955 to 0.690 | *t*(6) = -0.393, *p* = 0.708 |  |
| Age | 9 | -0.173 (1.098) | -2.771 to 2.424 | *t*(7) = -0.158, *p* = 0.879 | 0.007 (0.032) | -0.070 to 0.083 | *t*(7) = 0.205, *p* = 0.844 | *F*(1,7) = 0.042, *p* = 0.844 |
| Body mass | 9 | -0.565 (2.503) | -6.484 to 5.355 | *t*(7) = -0.226, *p* = 0.828 | 0.009 (0.036) | -0.075 to 0.093 | *t*(7) = 0.246, *p* = 0.813 | *F*(1,7) = 0.060, *p* = 0.813 |
| Height | 8 | 1.765 (7.830) | -17.396 to 20.925 | *t*(6) = 0.225, *p* = 0.829 | -0.010 (0.045) | -0.119 to 0.099 | *t*(6) = -0.221, *p* = 0.833 | *F*(1,6) = 0.049, *p* = 0.833 |
| Initial VO_2_max | 9 | 0.515 (1.488) | -3.003 to 4.033 | *t*(7) = 0.346, *p* = 0.739 | -0.008 (0.027) | -0.071 to 0.054 | *t*(7) = -0.315, *p* = 0.762 | *F*(1,7) = 0.099, *p* = 0.762 |
| Performance level | 9 |  |  |  |  |  |  | *F*(2,6) = 0.265, *p* = 0.776 |
| Moderately trained | 4 | 0.125 (0.232) | -0.433 to 0.693 | *t*(6) = 0.537, *p* = 0.610 |  |  |  |  |
| Well trained | 3 | 0.084 (0.265) | -0.566 to 0.733 | *t*(6) = 0.316, *p* = 0.763 | -0.041 (0.353) | -0.904 to 0.822 | *t*(6) = -0.116, *p* = 0.911 |  |
| Highly trained | 2 | -0.165 (0.336) | -0.989 to 0.658 | *t*(6) = -0.492, *p* = 0.640 | -0.290 (0.409) | -1.290 to 0.710 | *t*(6) = -0.710, *p* = 0.504 |  |
| Strength training experience | 6 |  |  |  |  |  |  | *F*(1,4) = 0.004, *p* = 0.952 |
| No | 5 | -0.054 (0.205) | -0.622 to 0.514 | *t*(4) = -0.265, *p* = 0.804 |  |  |  |  |
| Yes | 1 | -0.088 (0.482) | -1.426 to 1.250 | *t*(4) = -0.182, *p* = 0.865 | -0.033 (0.524) | -1.487 to 1.42 | *t*(4) = -0.064, *p* = 0.952 |  |
| Strength training intervention |  |  |  |  |  |  |  |  |
| Weeks | 9 | 0.321 (0.602) | -1.104 to 1.745 | *t*(7) = 0.532, *p* = 0.611 | -0.032 (0.068) | -0.192 to 0.129 | *t*(7) = -0.467, *p* = 0.655 | *F*(1,7) = 0.218, *p* = 0.655 |
| Sessions per week | 9 | 0.987 (1.053) | -1.502 to 3.476 | *t*(7) = 0.938, *p* = 0.380 | -0.444 (0.493) | -1.611 to 0.722 | *t*(7) = -0.901, *p* = 0.398 | *F*(1,7) = 0.811, *p* = 0.398 |
| Total sessions | 9 | 0.538 (0.579) | -0.831 to 1.906 | *t*(7) = 0.929, *p* = 0.384 | -0.027 (0.031) | -0.100 to 0.046 | *t*(7) = -0.877, *p* = 0.410 | *F*(1,7) = 0.769, *p* = 0.410 |

In the subgroup analysis (categorical variables), the first variable of the category was considered as the reference. n groups: number of experimental groups; SE, standard error; CI, confidence Interval; df, degree freedom.

**Electronic Supplementary Material Table S7**

**Article title:** The effect of strength training methods on middle- and long-distance runners’ athletic performance: A systematic review with meta-analysis.

**Author names:** Cristian Llanos-Lagos, Rodrigo Ramirez-Campillo, Jason Moran, Eduardo Sáez de Villarreal

**Affiliation and e-mail of the corresponding author:**

MSc. Cristian Llanos-Lagos

Universidad Pablo de Olavide

Physical Performance Sports Research Center

41704 Sevilla

Spain

Email: cfllalag@alu.upo.es

ORCID: 0009-0003-6906-4977

**Table S7.** Results of meta-regression and subgroup analyses in search of possible moderators of plyometric training on maximum metabolic steady state.

| **Plyometric training** | *n* groups | *β_0_* Hedges' *g* (SE) | CI 95% | *t_0_*(*df*)*, p* value | *β_1_* Hedges' *g* (SE) | CI 95% | *t_1_*(*df*)*, p* value | *F*(*df_1_, df_2_*)*, p* value |
| --- | --- | --- | --- | --- | --- | --- | --- | --- |
| Subject characteristics |  |  |  |  |  |  |  |  |
| Sex | 10 |  |  |  |  |  |  | *F*(2,7) = 1.457, *p* = 0.296 |
| Male | 6 | 0.063 (0.166) | -0.330 to 0.457 | *t*(7) = 0.380, *p* = 0.715 |  |  |  |  |
| Female | 2 | -0.523 (0.360) | -1.374 to 0.328 | *t*(7) = -1.453, *p* = 0.189 | -0.586 (0.397) | -1.524 to 0.351 | *t*(7) = -1.479, *p* = 0.183 |  |
| Male-Female | 2 | 0.250 (0.305) | -0.471 to 0.972 | *t*(7) = 0.821, *p* = 0.439 | 0.187 (0.347) | -0.635 to 1.009 | *t*(7) = 0.538, *p* = 0.607 |  |
| Age | 10 | 0.086 (0.168) | -0.302 to 0.473 | *t*(8) = 0.510, *p* = 0.624 | -0.001 (0.002) | -0.005 to 0.003 | *t*(8) = -0.689, *p* = 0.510 | *F*(1,8) = 0.475, *p* = 0.510 |
| Body mass | 10 | 0.086 (0.163) | -0.291 to 0.463 | *t*(8) = 0.526, *p* = 0.613 | -0.001 (0.001) | -0.002 to 0.001 | *t*(8) = -0.752, *p* = 0.474 | *F*(1,8) = 0.566, *p* = 0.474 |
| Height | 8 | -0.662 (0.995) | -3.097 to 1.772 | *t*(6) = -0.666, *p* = 0.530 | 0.003 (0.006) | -0.011 to 0.018 | *t*(6) = 0.564, *p* = 0.593 | *F*(1,6) = 0.318, *p* = 0.593 |
| Initial VO_2_max | 10 | -0.920 (0.722) | -2.584 to 0.744 | *t*(8) = -1.275, *p* = 0.238 | 0.017 (0.013) | -0.012 to 0.046 | *t*(8) = 1.322, *p* = 0.223 | *F*(1,8) = 1.748, *p* = 0.223 |
| Performance level | 10 |  |  |  |  |  |  | *F*(2,7) = 0.455, *p* = 0.652 |
| Moderately trained | 5 | -0.073 (0.211) | -0.572 to 0.426 | *t*(7) = -0.346, *p* = 0.739 |  |  |  |  |
| Well trained | 2 | -0.098 (0.290) | -0.783 to 0.588 | *t*(7) = -0.338, *p* = 0.745 | -0.025 (0.359) | -0.873 to 0.823 | *t*(7) = -0.069, *p* = 0.947 |  |
| Highly trained | 3 | 0.185 (0.222) | -0.341 to 0.711 | *t*(7) = 0.832, *p* = 0.433 | 0.258 (0.307) | -0.467 to 0.983 | *t*(7) = 0.842, *p* = 0.428 |  |
| Strength training intervention |  |  |  |  |  |  |  |  |
| Weeks | 10 | 0.401 (0.751) | -1.331 to 2.134 | *t*(8) = 0.534, *p* = 0.608 | -0.053 (0.101) | -0.287 to 0.181 | *t*(8) = -0.520, *p* = 0.617 | *F*(1,8) = 0.270, *p* = 0.617 |
| Sessions per week | 10 | 0.331 (0.424) | -0.646 to 1.309 | *t*(8) = 0.782, *p* = 0.457 | -0.185 (0.236) | -0.729 to 0.360 | *t*(8) = -0.782, *p* = 0.457 | *F*(1,8) = 0.612, *p* = 0.457 |
| Total sessions | 10 | 0.364 (0.417) | -0.596 to 1.325 | *t*(8) = 0.874, *p* = 0.408 | -0.028 (0.032) | -0.103 to 0.046 | *t*(8) = -0.881, *p* = 0.404 | *F*(1,8) = 0.776, *p* = 0.404 |

In the subgroup analysis (categorical variables), the first variable of the category was considered as the reference. n groups: number of experimental groups; SE, standard error; CI, confidence Interval; df, degree freedom.

**Electronic Supplementary Material Table S8**

**Article title:** The effect of strength training methods on middle- and long-distance runners’ athletic performance: A systematic review with meta-analysis.

**Author names:** Cristian Llanos-Lagos, Rodrigo Ramirez-Campillo, Jason Moran, Eduardo Sáez de Villarreal

**Affiliation and e-mail of the corresponding author:**

MSc. Cristian Llanos-Lagos

Universidad Pablo de Olavide

Physical Performance Sports Research Center

41704 Sevilla

Spain

Email: cfllalag@alu.upo.es

ORCID: 0009-0003-6906-4977

**Table S8.** Results of meta-regression and subgroup analyses in search of possible moderators of high load training on running performance.

| **High load training** | *n* groups | *β_0_* Hedges' *g* (SE) | CI 95% | *t_0_*(*df*)*, p* value | *β_1_* Hedges' *g* (SE) | CI 95% | *t_1_*(*df*)*, p* value | *F*(*df_1_, df_2_*)*, p* value |
| --- | --- | --- | --- | --- | --- | --- | --- | --- |
| Subject characteristics |  |  |  |  |  |  |  |  |
| Sex | 8 |  |  |  |  |  |  | *F*(2,5) = 1.082, *p* = 0.407 |
| Male | 4 | -0.665 (0.240) | -1.281 to -0.048 | ***t*(5) = -2.772, *p* = 0.039** |  |  |  |  |
| Female | 2 | -0.071 (0.326) | -0.911 to 0.768 | *t*(5) = -0.218, *p* = 0.836 | 0.593 (0.405) | -0.448 to 1.635 | *t*(5) = 1.465, *p* = 0.203 |  |
| Male-Female | 2 | -0.511 (0.362) | -1.442 to 0.420 | *t*(5) = -1.411, *p* = 0.217 | 0.154 (0.434) | -0.963 to 1.270 | *t*(5) = 0.354, *p* = 0.738 |  |
| Age | 8 | -1.707 (0.900) | -3.909 to 0.496 | *t*(6) = -1.896, *p* = 0.107 | 0.041 (0.030) | -0.031 to 0.114 | *t*(6) = 1.401, *p* = 0.211 | *F*(1,6) = 1.962, *p* = 0.211 |
| Body mass | 8 | -0.661 (2.186) | -6.011 to 4.688 | *t*(6) = -0.302, *p* = 0.773 | 0.003 (0.032) | -0.076 to 0.081 | *t*(6) = 0.088, *p* = 0.933 | *F*(1,6) = 0.008, *p* = 0.933 |
| Height | 8 | 4.213 (5.345) | -8.867 to 17.293 | *t*(6) = 0.788, *p* = 0.461 | -0.027 (0.031) | -0.102 to 0.048 | *t*(6) = -0.876, *p* = 0.415 | *F*(1,6) = 0.768, *p* = 0.415 |
| Initial VO_2_max | 8 | 1.092 (1.242) | -1.947 to 0.879 | *t*(6) = 0.879, *p* = 0.413 | -0.028 (0.022) | -0.082 to 0.026 | *t*(6) = -1.269, *p* = 0.251 | *F*(1,6) = 1.610, *p* = 0.251 |
| Performance level | 8 |  |  |  |  |  |  | *F*(2,5) = 1.223, *p* = 0.370 |
| Moderately trained | 2 | -0.303 (0.364) | -1.237 to 0.632 | *t*(5) = -0.832, *p* = 0.443 |  |  |  |  |
| Well trained | 4 | -0.320 (0.234) | -0.922 to 0.281 | *t*(5) = -1.368, *p* = 0.229 | -0.018 (0.432) | -1.129 to 1.094 | *t*(5) = -0.041, *p* = 0.969 |  |
| Highly trained | 2 | -0.931 (0.341) | -1.809 to -0.041 | ***t*(5) = -2.728, *p* = 0.041** | -0.629 (0.499) | -1.911 to 0.653 | *t*(5) = -1.260, *p* = 0.263 |  |
| Strength training intervention |  |  |  |  |  |  |  |  |
| Weeks | 8 | -1.276 (0.897) | -3.472 to 0.920 | *t*(6) = -1.422, *p* = 0.205 | 0.097 (0.106) | -0.163 to 0.358 | *t*(6) = 0.916, *p* = 0.395 | *F*(1,6) = 0.839, *p* = 0.395 |
| Sessions per week | 8 | 0.561 (0.854) | -1.528 to 2.649 | *t*(6) = 0.657, *p* = 0.536 | -0.433 (0.352) | -1.295 to 0.428 | *t*(6) = -1.231, *p* = 0.265 | *F*(1,6) = 1.514, *p* = 0.265 |
| Total sessions | 8 | -0.299 (0.632) | -1.846 to 1.248 | *t*(6) = -0.473, *p* = 0.653 | -0.009 (0.031) | -0.083 to 0.066 | *t*(6) = -0.281, *p* = 0.788 | *F*(1,6) = 0.079, *p* = 0.788 |

In the subgroup analysis (categorical variables), the first variable of the category was considered as the reference. n groups: number of experimental groups; SE, standard error; CI, confidence Interval; df, degree freedom. Results in bold represent a significant effect (α = 0.05).

**Electronic Supplementary Material Table S9**

**Article title:** The effect of strength training methods on middle- and long-distance runners’ athletic performance: A systematic review with meta-analysis.

**Author names:** Cristian Llanos-Lagos, Rodrigo Ramirez-Campillo, Jason Moran, Eduardo Sáez de Villarreal

**Affiliation and e-mail of the corresponding author:**

MSc. Cristian Llanos-Lagos

Universidad Pablo de Olavide

Physical Performance Sports Research Center

41704 Sevilla

Spain

Email: cfllalag@alu.upo.es

ORCID: 0009-0003-6906-4977

**Table S9.** Results of meta-regression and subgroup analyses in search of possible moderators of plyometric training on running performance.

| **Plyometric training** | *n* groups | *β_0_* Hedges' *g* (SE) | CI 95% | *t_0_*(*df*)*, p* value | *β_1_* Hedges' *g* (SE) | CI 95% | *t_1_*(*df*)*, p* value | *F*(*df_1_, df_2_*)*, p* value |
| --- | --- | --- | --- | --- | --- | --- | --- | --- |
| Subject characteristics |  |  |  |  |  |  |  |  |
| Sex | 14 |  |  |  |  |  |  | *F*(1,12) = 0.450, *p* = 0.515 |
| Male | 9 | -0.279 (0.146) | -0.598 to 0.040 | *t*(12) = -1.906, *p* = 0.081 |  |  |  |  |
| Male-Female | 5 | -1.140 (0.146) | -0.459 to 0.179 | *t*(12) = -0.958, *p* = 0.357 | 0.139 (0.207) | -0.312 to 0.590 | *t*(12) = 0.670, *p* = 0.515 |  |
| Age | 14 | 0.384 (0.633) | -0.955 to 1.763 | *t*(12) = 0.607, *p* = 0.555 | -0.020 (0.021) | -0.067 to 0.026 | *t*(12) = -0.951, *p* = 0.360 | *F*(1,12) = 0.905, *p* = 0.360 |
| Body mass | 14 | 1.026 (1.421) | -2.070 to 4.122 | *t*(12) = 0.772, *p* = 0.484 | -0.018 (0.021) | -0.064 to 0.027 | *t*(12) = -0.872, *p* = 0.400 | *F*(1,12) = 0.760, *p* = 0.400 |
| Height | 10 | 4.471 (6.852) | -11.329 to 20.271 | *t*(8) = 0.653, *p* = 0.532 | -0.027 (0.040) | -0.118 to 0.064 | *t*(8) = -0.684, *p* = 0.514 | *F*(1,8) = 0.467, *p* = 0.514 |
| Initial VO_2_max | 10 | 0.429 (1.047) | -1.986 to 2.845 | *t*(8) = 0.410, *p* = 0.693 | -0.010 (0.018) | -0.051 to 0.031 | *t*(8) = -0.568, *p* = 0.586 | *F*(1,8) = 0.323, *p* = 0.586 |
| Performance level | 14 |  |  |  |  |  |  | *F*(2,11) = 0.099, *p* = 0.907 |
| Moderately trained | 5 | -0.232 (0.163) | -0.592 to 0.128 | *t*(11) = -1.419, *p* = 0.184 |  |  |  |  |
| Well trained | 5 | -0.137 (0.194) | -0.565 to 0.290 | *t*(11) = -0.706, *p* = 0.495 | 0.095 (0.254) | -0.464 to 0.654 | *t*(11) = 0.373, *p* = 0.716 |  |
| Highly trained | 4 | -0.246 (0.184) | -0.652 to 0.159 | *t*(11) = -1.337, *p* = 0.208 | -0.014 (0.246) | -0.557 to 0.528 | *t*(11) = -0.059, *p* = 0.954 |  |
| Strength training experience | 9 |  |  |  |  |  |  | *F*(1,7) = 0.002, *p* = 0.970 |
| No | 8 | -0.185 (0.122) | -0.472 to 0.103 | *t*(7) = -1.520, *p* = 0.172 |  |  |  |  |
| Yes | 1 | -0.204 (0.463) | -1.297 to 0.890 | *t*(7) = -0.440, *p* = 0.673 | -0.019 (0.478) | -1.150 to 1.112 | *t*(7) = -0.039, *p* = 0.970 |  |
| Strength training intervention |  |  |  |  |  |  |  |  |
| Weeks | 14 | -0.309 (0.445) | -1.280 to 0.661 | *t*(12) = -0.694, *p* = 0.501 | 0.013 (0.055) | -0.108 to 0.133 | *t*(12) = 0.230, *p* = 0.822 | *F*(1,12) = 0.053, *p* = 0.822 |
| Sessions per week | 14 | -0.264 (0.296) | -0.909 to 0.382 | *t*(12) = -0.890, *p* = 0.391 | 0.027 (0.140) | -0.277 to 0.331 | *t*(12) = 0.195, *p* = 0.849 | *F*(1,12) = 0.038, *p* = 0.849 |
| Total sessions | 14 | -0.246 (0.224) | -0.734 to 0.242 | *t*(12) = -1.099, *p* = 0.293 | 0.002 (0.012) | -0.025 to 0.029 | *t*(12) = 0.184, *p* = 0.857 | *F*(1,12) = 0.034, *p* = 0.857 |

In the subgroup analysis (categorical variables), the first variable of the category was considered as the reference. n groups: number of experimental groups; SE, standard error; CI, confidence Interval; df, degree freedom.

**Electronic Supplementary Material Figure S1**

**Article title:** The effect of strength training methods on middle- and long-distance runners’ athletic performance: A systematic review with meta-analysis.

**Author names:** Cristian Llanos-Lagos, Rodrigo Ramirez-Campillo, Jason Moran, Eduardo Saez de Villarreal

**Affiliation and e-mail of the corresponding author:**

MSc. Cristian Llanos-Lagos

Universidad Pablo de Olavide

Physical Performance Sports Research Center

s41704 Sevilla

Spain

Email: cfllalag@alu.upo.es

ORCID: 0009-0003-6906-4977

**Fig S1**. Funnel plot of studies of different strength training methods that analysed related outcomes.


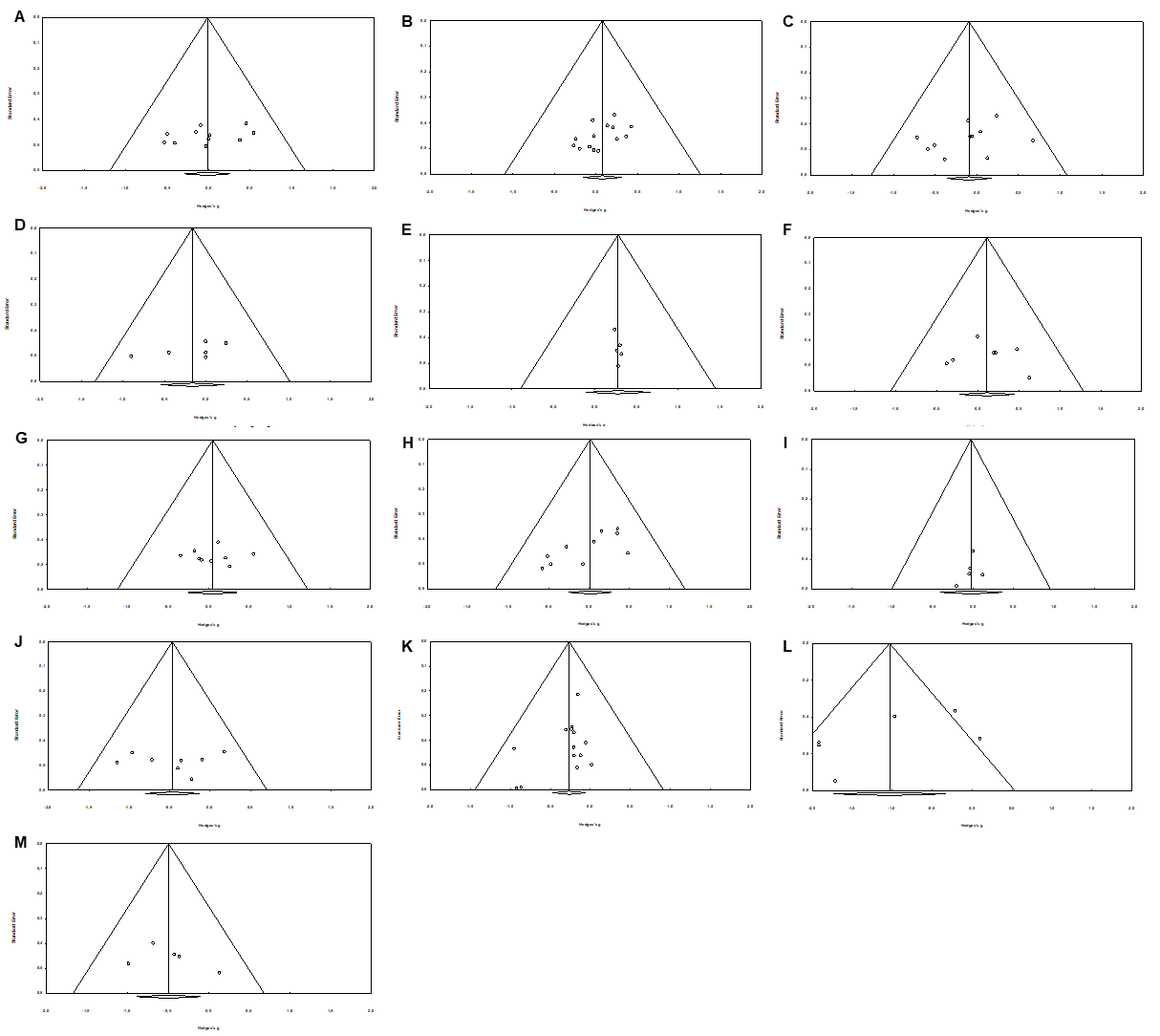


Maximal oxygen uptake (VO_2_max; A, high load training; B, plyometric training; C, combined training), velocity at VO_2_max (D, high load training; E, plyometric training; F, combined training), maximum metabolic steady state (G, high load training; H, plyometric training; I, combined training), running performance (J, high load training; K, plyometric training; L, combined training) and, sprint capacity (M, combined training) plots.
